# Plant-Derived Exosomes: Nano-Inducers of Cross-Kingdom Regulations

**DOI:** 10.3390/ph18071005

**Published:** 2025-07-04

**Authors:** Touseef Ur Rehman, Huiliang Li, Maria Martuscelli, Francesca Aiello, Luigi Esposito, Kamran Ashraf, Meijin Guo, Ali Mohsin

**Affiliations:** 1 State Key Laboratory of Bioreactor Engineering, East China University of Science and Technology, 130 Meilong Road, Shanghai 200237, China; touseef1992@gmail.com (T.U.R.); yuanzhi@eastgarden.com (H.L.); y10210214@mail.ecust.edu.cn (K.A.); alimohsin@ecust.edu.cn (A.M.); 2 Department of Bioscience and Food, Agricultural and Environmental Technology, University of Teramo, Via Balzarini 1, 64100 Teramo, Italy; lesposito2@unite.it; 3 Department of Pharmacy, Health and Nutritional Sciences, University of Calabria, 87036 Rende, Italy; francesca.aiello@unical.it

**Keywords:** plant-derived exosomes, exosomes isolation, exosomes biogenesis, exosomes characterization, exosomes engineering, targeted therapies, cross-kingdom regulations

## Abstract

Exosomes are essential components produced by all cell types, originating from the endosomal pathway through the invagination of the cell membrane. Their unique physicochemical characteristics are crucial for various commercial applications. Typically, exosomes range in size from 50 to 200 nm. Exosomes derived from plant cells are larger than their animal cell counterparts and demonstrate a broader therapeutic potential. This review explores the promising research opportunities associated with plant-derived exosomes, summarizing studies on their biogenesis, characterization, isolation methods, and therapeutic applications. It also emphasizes the importance of targeted drug delivery and provides insights into engineering plant-derived exosomes with various drugs. Additionally, highlights of plant-derived exosomes as natural nano-inducers that facilitate inter-kingdom communication and cross-kingdom regulatory interactions are also elucidated herein. Henceforth, this study culminates in a multidimensional insight for innovative therapeutic strategies and biotechnological advancements in plant-derived exosome research.

## 1. Introduction

With the advancement of scientific nomenclature, the number of identified species of the Kingdom Plantae has increased significantly, reaching over 374,000 currently recognized species. Nevertheless, about 20,000 new plant species have been discovered. [1]. As a result, the scope of research and development in plant science is expanding each day. Historically, plants have been used extensively for the treatment of various diseases, and now the role of plants in therapeutic research is also well recognized for the treatment of inflammatory chronic diseases, respiratory illnesses, and even for the treatment of various forms of cancer [2]. Essential metabolites produced by cells are responsible for these therapeutic effects [3]. Furthermore, cellular organelles also contain the biochemical and bioinformatic constitution of the cell [4]. Some of these organelles are naturally released by the cell into the extracellular environment, known as extracellular vesicles, to facilitate cell functions [5]. Among these extracellular vesicles naturally released into the extracellular space are the exosomes [6].

At first, the word exosomes was used for DNA fragments from an external source that created associations with homologous chromosomes in a treated organism [7]. However, in succeeding publications by two different groups of scientists [8,9], reports of nano-sized extracellular vesicles in reticulocytes were quoted, which were later termed exosomes by Rose Johnstone, and their scope in terms of synthesis and importance initiated extensive scientific research [10]. In detail, exosomes are nano-sized cellular vesicles containing biological components from the parent cell, and the representative size range of exosomes is 50–200 nm, encapsulated in a lipid bilayer and further used for immunogenic responses [11]. Characteristically, exosomes are reported to have a float density in the range of 1.13 to 1.19 g mL^−1^ [12]. Furthermore, exosomes are spherical in shape [13]. Regarding functionality, it has been proposed that exosomes can also be used to remove cellular waste [14]. In addition, plant-derived exosomes differ from animal exosomes in their unique plant-specific cargo (lipids, proteins, metabolites), lower immunogenicity, and superior scalability [15].

Regarding cellular biogenesis, the generation of plant-derived exosomes starts from multivesicular bodies (MVBs), mainly due to cellular responses [16]. In the biosynthetic pathway, the endosomal sorting complex required for transport (ESCRT) plays a key role in the production of exosomes in the cell [17]. A similar synthetic pathway for plant-derived exosomes from MVBs has been reported in the case of *Catharanthus roseus* (L.), and these exosomes were resilient to high pH values and also displayed a higher level of stability in gastrointestinal fluids. In addition to these resistant properties, the exosomes contained important functional lipids and also several marker proteins [18].

In addition to these naturally secreted exosomes in the extracellular space, for scientific analysis and for use in the therapeutic industry, exosomes are isolated synthetically or mechanically by using various isolation methods [19]. Different isolation techniques, like polymer-based precipitation, ultrafiltration, and ultracentrifugation, are employed to isolate exosomes from numerous sources [20]. After isolation, exosomes are characterized. Therefore, accurate characterization is necessary for their effective use in scientific and therapeutic research [21].

Each type of eukaryotic cell produces exosomes that are released from the cells and are characterized by their biochemical composition and function [22]. In this regard, exosomes are analyzed by using a variety of approaches, including parameters for physiological evaluation, such as size, shape, and surface charge, as well as approaches for the biochemical and bioinformatic profiling of different biological components present in exosomes [23,24]. Regarding their bio-composition, the presence of some essential metabolites, such as sugars, alcohols, carboxylic acids, amino acids, amides, and enzymes, has been reported to be present in exosomes [25]. Moreover, exosomes also contain many cellular proteins, particularly syntenin-1 [26], and the exosome proteome composition demonstrates their efficacy against biotic and abiotic stress responses [27].

Additionally, the presence of nucleic acid content also represents exosome capacity in therapeutics [28]. It has also been suggested that the miRNA content of vesicles derived from plants has a pronounced effect on the human genome [29]. Moreover, the exosomes are also prospectively used for the diagnosis and treatment of viral diseases such as HBV, HCV, HIV, and SARS-CoV-2 infection [30]. It has been demonstrated that plant-derived exosomes can serve as drug delivery agents, play a significant role in the development of resistance to a particular disease, and even exhibit resistance against various pathogen attacks [31]. However, to utilize exosomes as targeted drug delivery agents, various tailoring methods are employed to load drugs onto exosomes [32]. Interestingly, along with these therapeutic features and properties, plant-derived exosomes can initiate cross-kingdom communication, resulting in the modulation of cross-kingdom regulation in mammalian cells [33].

Therefore, in this review, we have briefly considered the research potential of plant-derived exosomes, comprising previous research on characterization, biogenesis, isolation techniques, and therapeutic potential. Furthermore, we have presented targeted drug delivery for targeted therapies and have cited approaches to tailor drugs with plant-derived exosomes. The fact that we have integrated all of these factors into a single study is of utmost importance, as it clearly demonstrates that plant-derived exosomes modulate cross-kingdom regulation via cross-kingdom communication.

## 2. Characterization of Exosomes

Exosomes from different sources are characterized in various ways based on their distinct characteristics [34]. Regarding mammalian cells, the prospective impact of exosomes on disease identification and treatment has been well reported and extensively studied [35]. However, research on plant-derived exosomes is still in the initial phase. Since exosomes are primarily derived from natural sources, it is necessary to characterize them. Accordingly, there are various techniques currently employed to accomplish the characterization of exosomes [36]. Techniques such as scanning electron microscopy (SEM), transmission electron microscopy (TEM), zeta-sizer, and atomic force microscopy (AFM) are used to analyze the morphological characteristics [33,37,38]. Similarly, nano tracking analysis (NTA) for the size-dependent concentration analysis of exosomes is also used for characterization [39].

**Figure 1:**
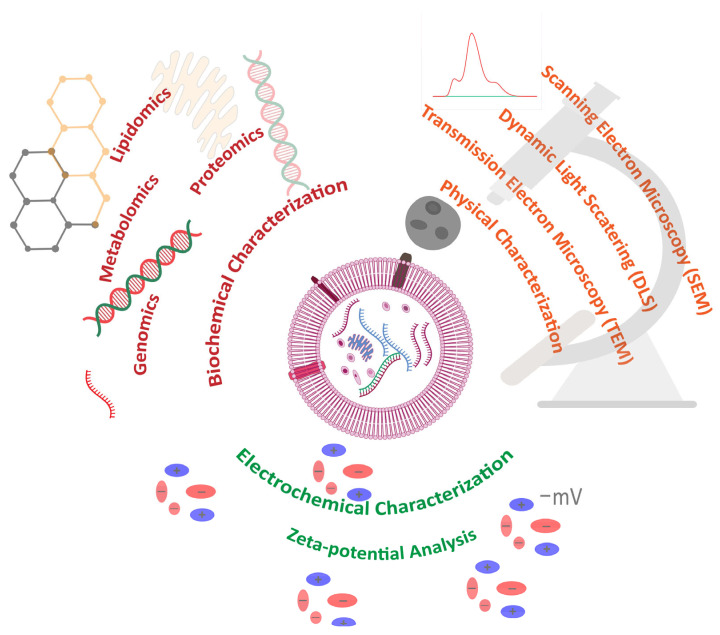
Commonly used methods for the physicochemical characterization of exosomes.

In terms of biochemical characterization, the biochemical composition of exosomes is exceptionally heterogeneous [40]. In point of fact, exosomes are bilipid membrane-bounded cellular organelles that are produced in the cell as a result of cell membrane invagination and contain cellular fluids such as proteins, nucleic acids, vitamins, secondary metabolites, and other essential cellular fluids [41]. In addition to exosomes, other biologically distinct particles are also secreted from cells [42]. Therefore, it is necessary to distinguish exosomes from other vesicles via biochemical profiling; many techniques have therefore been employed to analyze the lipid, protein, and metabolomic constituents of exosomes [24,43]. Approaches such as mass spectrometry (MS), Fourier-transform infrared spectroscopy (FTIR), enzyme-linked immunosorbent assay (ELISA), sulfo-phospho-vanillin (SPV) assay, next-generation sequencing (NGS), and polymerase chain reaction (PCR) are used for biochemical and bioinformatic compositional analysis [24]. In the future, the characterization will enable the effective use of exosomes as biomarkers for various diseases, as well as for disease prevention and treatment [44]. Commonly used methods for the physicochemical characterization of exosomes are shown in Figure 1.

### 2.1. Physical Characterization of Exosomes

**Figure 2:**
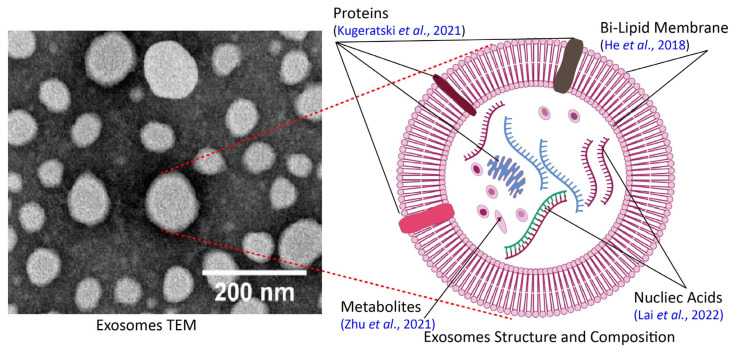
Structural morphology of plant-derived exosomes and biochemical composition [24,26,41,64].

The exosomes produced by mammalian cells have been reported to have a circular or cup-shaped topography when analyzed by transmission electron microscopy (TEM), and the diameter of these exosomes usually ranges from 30 to 100 nm [45,46]. Regarding exosomes from plant sources, a study on the isolation and characterization of exosomes from beetroot (*Beta vulgaris*) through atomic force microscopy (AFM) and field emission scanning electron microscopy (FE-SEM) reported that the exosomes were round in shape with relatively smaller size, i.e., 50 nm or less [47]. Meanwhile, the exosomes produced by plant cell sources of green tea (*Camellia sinensis*) and ginseng (*Panax ginseng*) after isolation were further analyzed by cryo-TEM, and are also reported to be round with a lipid bilayer, but their sizes on average are 159 nm and 149 nm, respectively, whereas exosomes isolated from cica (*Cantella asiatica*) and purslane (*Portulaca oleracea*) have an average size of 167 nm and 157 nm [48]. On the other hand, some exosomes from various plant sources have a cup-shaped appearance, with a relatively larger size range of 50–500 nm [49]. Moreover, it has been reported that the size of exosomes like nanoparticles isolated from ginger (*Zingiber Officinale*) varies with respect to the procedure used, i.e., their reported average sizes from different sources are 403 nm (by ultracentrifugation method), 365 nm (by PEG 8% precipitation technique), 304 nm (by PEG 10% precipitation technique), 263 nm (by PEG 12% precipitation technique), and 252 nm (by PEG 15% precipitation technique) [50]. This shows that exosomes isolated from plant sources are larger in size than those of exosomes isolated from animal sources. Table 1 illustrates a variety of sizes and shapes from various plant sources in light of previously published data, and the structural morphology of exosomes is represented in Figure 2. (TEM [18]).

**Table 1:** Characterization of plant-derived exosomes.

Plants	Size	Shape	Zeta-Potential	Reference
*Catharanthus roseus* (L.)	50 and 100 nm	Rounded hollow vesicle shape	−21.8 mV	[18]
*Artemisia annua* (L.)	106.9 nm (Average)	Spherical	−22.5 mV	[33]
Asian ginseng (*P. ginseng*)	241.1 ± 3.8 nm (Analyzed Further) 144.1 ± 2.8 nm 340.1 ± 15.9 nm	Cup-shaped	−27.4 ± 0.45 mV	[37]
	105.8 nm (Average)	Spherical	−20.7 mV	[51]
	344.8 nm (Average)	Spherical	−25.4 mV	[52]
	50–150nm	Spherical	−20.61 mV (Ultracentrifugation) −28.88mV (ExoQuick) −29.54 mV (Combination of Exo-Quick and Ultracentrifugation)	[53]
	146.5 nm (Average)	Cup-shaped	−19.2 mV	[54]
*Arabidopsis thaliana*	50–150 nm	Spherical	−17.1 mV (Ultracentrifugation) −21.3 mV (ExoQuick) −25.9 mV (Combination of EXO-Quick and Ultracentrifugation)	[53]
Garlic (Allium sativum *Linn*)	100 to 300 nm	Sphere-shaped	−7.8 mV	[55]
	100–300 nm	Sphere-shaped	−8 mV	[56]
Curcumae Rhizoma (*Curcuma longa* L.)	100–180 nm	Bowl-shaped	−20.90 mV	[57]
Cabbage (*Brassica oleracea*)	100 nm (Average)	Spherical	−14.8 mV Cabbage −15.2 mV Red Cabbage	[58]
Tartary buckwheat (*Fagopyrum tataricum*)	30–200 nm	Round- or Cup-shaped	−7.2 mV	[59]
Dandelion (*Taraxacum officinale*)	142.5 nm (Average)	Disk-like or Spherical	−41.83 mV	[60]
Tomato (*Solanum lycopersicum*)	140 to 170 nm	Spherical or oval-shaped	−24 mV (Approx)	[61]
Grapefruit (*Citrus paradise)*	86 to 125 nm	Spherical or oval-shaped	−10 mV	[61]
*Portulaca oleracea* (L.)	160 nm (Average)	Round	−31.4 mV	[62]
Turmeric (*Curcuma longa*)	178 nm (Average)	Saucer-shaped	−21.7 mV	[63]

### 2.2. Electrochemical Characterization of Exosomes

Exosomes show great promise as biomarkers for diagnosing and treating diseases like cancer, and as biological carriers for delivering drugs and bio-nutrients, where the zeta-potential plays a critical role in enabling these therapeutic effects [65,66,67]. Characterization by surface charge (zeta-potential) reflects their stability and propensity for cellular uptake [68]. The zeta potential of exosomes from different sources may differ from each other; thus, zeta potential is an essential factor for the reliable characterization of exosomes [69].

In the case of grape-derived exosomes, the average zeta potential of nanovesicles has been reported within a range of −26.3 mV to −8.14 mV [70]. While the zeta potential value of ginger-derived exosomes, like nanoparticles, has been reported from −24.6 mV to −29.7 mV [71], in another research study, the ginger zeta potential is reported as −12 mV [72]. Here, a question comes to mind: Why is there a variation in the zetapotential of similar nanovesicles that are being isolated from a similar source? The answer is that zetapotential can vary based on the pH of the solutions, the concentration of components in the formulation, and their conductivity [73]. It has also been reported that the zetapotential value of replicates showed considerable difference with respect to the method being used for the isolation of ginger-derived exosomes, while the recorded values of zeta potential are −25.7 mV by ultracentrifugation method, −25.5 by PEG 8% (precipitation technique), −25.1 by PEG 10% (precipitation technique), −21.4 by PEG 12% (precipitation technique), and −21.2 by PEG 15% (precipitation technique) [50]. This clearly illustrates that isolated exosomes from all eukaryotic sources have zeta potential values differing from each other, and the values can also vary regarding the method being used for isolation, as well as other associated factors, as mentioned above. Many researchers have reported the zeta potential of various exosomes from distinct plant sources, as described in Table 1.

### 2.3. Biochemical Characterization of Exosomes

Different exosomes carry different biological components, leading to different uses in therapeutic approaches [74]. Profiling the lipid content of exosomes is critical, as lipid content can regulate the production and therapeutic propensity of exosomes [75]. There are several methods being employed to analyze lipid content in exosomes, such as MS (mass spectrometry), HPLC–MS/MS (high-performance liquid chromatography–tandem mass spectrometry), MALDI–MS (matrix-assisted laser desorption/ionization), UHPLC/MS (ultrahigh-performance liquid chromatography–mass spectrometry), and UHPSFC/MS (ultrahigh-performance supercritical fluid chromatography–mass spectrometry) [76,77,78]. Due to the presence of a bilipid membrane, exosomes have significant lipid concentrations. In addition, sphingolipids, glycosphingolipids, phospholipids, ceramides, cholesterol, and phosphatidylserine are the major lipid components [79]. It has also been reported that more than 30% of ether phospholipids are present among other lipid types, and it has also been hypothesized that the pH of the isolation environment and the lipophilic properties of compounds may influence the composition of plant-derived exosomes like nanoparticles (PENPs) [18].

Analysis of protein concentration is also essential for exosome research. Protein profiling of exosomes is carried out by various methods, including MS (mass spectrometry)-based proteomic analysis [80] and super-SILAC (super-stable isotope labeling with amino acids in cell culture) [26]. Different types of proteins are reported to be present in exosomes, particularly ESCRT protein complexes and other types such as heat shock proteins for shielding against heat stress and cytoskeletal proteins for maintaining structural stability [81].

Furthermore, metabolomics is essential for the profiling of small molecules and aids in predicting the possible outcomes of biochemical reactions [82]. Methods like UHPLC/MS carry out metabolomics analyses [83]. In a recent study, 196 metabolites were present in exosomes, mainly consisting of benzene, amino acids, lipids, fatty acids, organic acids, fatty acyls, and carbohydrates [64].

**Figure 3:**
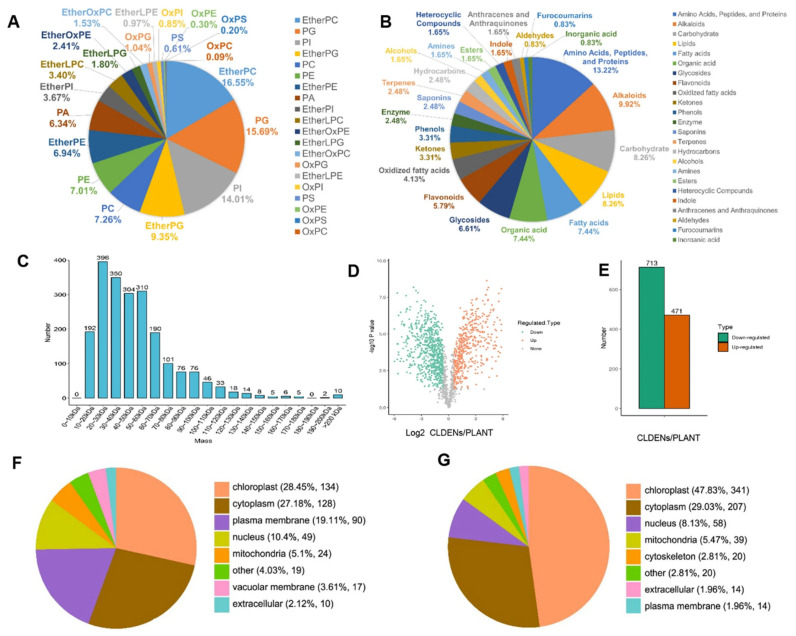
Compositional analysis of CLDENs. (**A**) Lipidomic analysis of CLDENs. (**B**) Metabolomic analysis of CLDENs. (**C**) Molecular weight distribution of the identified proteins in the CLDENs group. (**D**) Volcano plot of proteomic analysis. (**E**) The number of differentially up-regulated and down-regulated proteins. (**F**,**G**) Subcellular localization of differentially expressed proteins, up-regulated proteins (**F**) and down-regulated proteins (**G**).

Analyzing and quantifying the presence of genetic components, such as RNA, in exosomes is an essential factor in scientific and therapeutic research on exosomes (80). Nucleic acid contents, such as mitochondrial DNA, and RNA derivatives, such as miRNA, circRNA, mRNA, ncRNA, and lncRNA, have also been reported to be present in exosomes [84]. In addition, the commonly used method to detect genetic components in exosomes is qRT-PCR (quantitative real-time polymerase chain reaction) analysis [85]. Therefore, biochemical analysis and the quantification of biotic components are important for the clinical and therapeutic use of plant-derived exosomes. The biochemical composition analysis of *Catharanthus roseus* (L.)-derived exosomes, like nanoparticles, is shown in Figure 3 [18].

### 2.4. Characterization of Exosomes Based on Source

Exosomes are produced in almost all types of cells, and their role depends on their source [86]. Exosomes from different sources have different traits [45]. Exosomes isolated from cancer cells exert immune-activating and immunosuppressive functions in cells and are used as biomarkers [87]. Brain cell-derived exosomes have an intercellular communication role and have been used in cell therapy for the treatment of stroke and traumatic brain injury [88]. Meanwhile, the liver cell-derived exosomes responsible for pathogenesis in the liver are exploited as biomarkers for liver-related diseases [89]. Similarly, cell-derived exosomes mediate immune responses that improve the functionality of antigen-presenting cells and boost the proliferative response of T-cells [90]. Bone cell-derived exosomes are prospectively used for therapeutic approaches related to bone homeostasis [91]. These are a few examples of animal cell-derived exosomes that can be used to better understand the therapeutic potential of exosomes based on source.

However, due to their abundance in the Kingdom Plantae, research on their isolation, physicochemical characterization, and therapeutic use is still at the initial stage. In plants, extracellular vesicles have been isolated from various plant parts, i.e., leaves, fruits, sap, stem, seeds, and roots [92], as well as from in vitro-grown cultures [93]. Different research groups have conducted several studies to use different plant sources, i.e., *Betula pubescens*, *Picea abies*, and *Populus balsamifera*, for checking the presence of exosomes in the phloem and xylem of woody plants; it is reported that exosomes were present in these plant tissues, and their average size ranges were 110  ±  10 nm, 97  ±  12 nm, and 107  ±  12 nm, respectively [94]. Furthermore, exosomes derived from leaves of *Catharanthus roseus* (L.), and from two different cell culture sources of the same plant, showed variation in particle size and surface charge, along with variable therapeutic potential; furthermore, the quantity of isolated exosomes in cases of cell cultures is reported to be relatively greater in contrast to the exosomes isolated from leaves [18]. Similarly, coconut-derived exosomes from two different sources, i.e., coconut water and coconut milk, exhibited characteristically different size ranges. The average size of vesicles derived from coconut water was 59.72 nm, and the average size of vesicles derived from coconut milk was approximately 100.40 nm [29].

**Figure 4:**
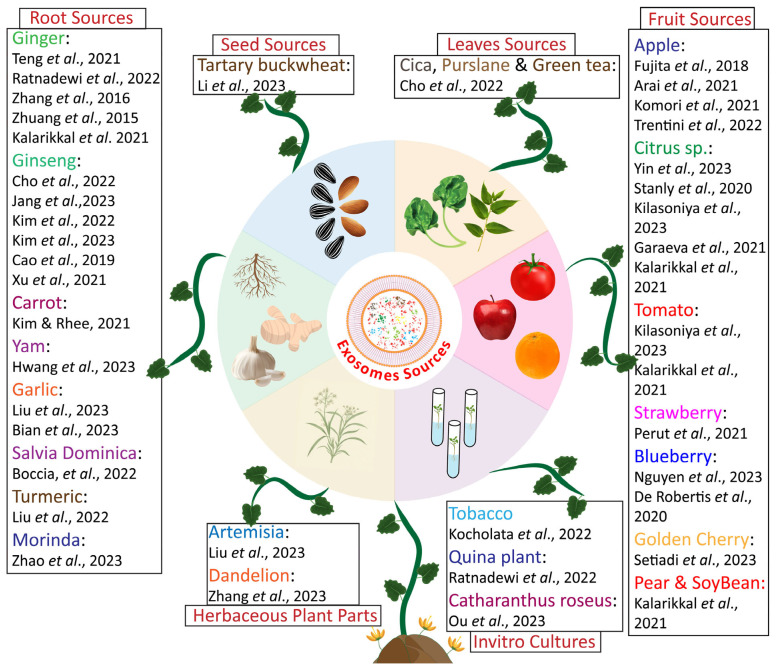
Sources of plant-derived exosomes in recent therapeutic studies [18,33,37,48,51,52,53,54,55,56,59,60,61,63,69,71,72,93,96,97,98,99,100,101,102,103,104,105,106,107,108,109,110,111,112,113].

Moreover, the isolation of exosomes from various plant sources has initiated a new trend for cost-effective therapeutic approaches by using exosomes, which will be briefly discussed in a later section. Whereas here we cite a comprehensive study for the understanding of advanced therapeutic approaches for plant-derived exosome usage in therapeutics, in this study, exosomes from several fruit and vegetable sources, i.e., mango (*Mangifera indica* L.), asparagus (*Asparagus officinalis*), orange (*Citrus sinensis* (L.) Osbeck), cherry (*Prunus avium* L.), grape (*Vitis vinifera* L.), grapefruit (*Citrus paradisi*), kiwi (*Actinidia chinensis*, lemon (*Citrus limon* (L.) Osbeck), papaya (*Carica papaya*), tomato (*Solanum Lycopersicum* L.), blood orange (*Citrus sinensis* (L.) Osbeck “Blood Orange”), and bergamot (Citrus × bergamia Risso and Poit.), were isolated together, resulting in an Exocomplex^®^ which exhibited high antioxidant activity and has been deemed fit for oral consumption [95]. These data demonstrated the therapeutic potential of plant-derived exosomes depending on the source cell. Furthermore, exosomes isolated and characterized from different plant sources in different reported studies are presented in Figure 4.

## 3. Biogenesis of Plant-Derived Exosomes

Exosomes are considered endosomal-originated organelles; putatively, their endosomal nature shows that they are associated with cell response, and exosomes may be produced as a response to a pathogen attack, and also for cell-to-cell communication [114]. Recently, research on cancer cells has shown that they exhibit aggressiveness in response to extracellular matrix (ECM) stiffening, and it has also been reported that such stress can stimulate the production and release of exosomes, and it also promotes uncontrolled cell division [115,116].

**Figure 5:**
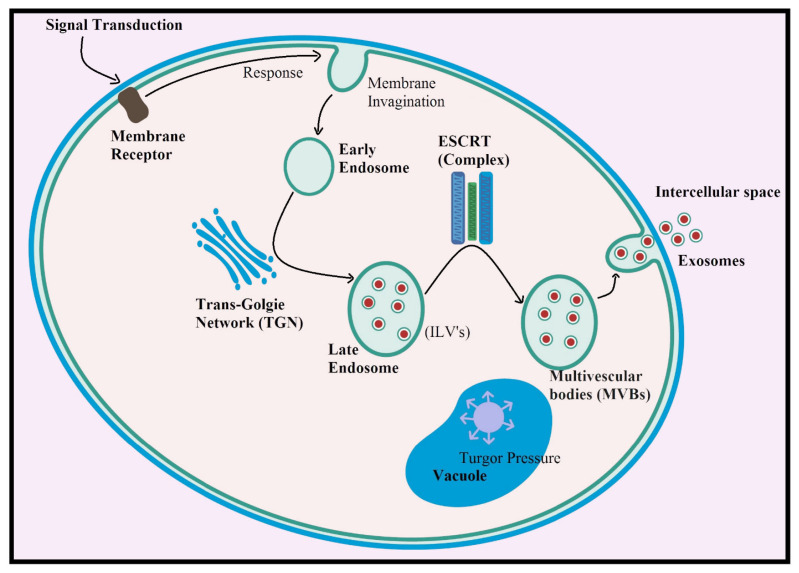
Proposed biogenesis pathway of exosomes in plant cells.

Since plant cells are eukaryotic in origin and use the same pathways as animal cells, the conclusion drawn from the studies, as mentioned above, indicates that exosome production begins in plant cells as a biological response to stress or to induce cell responses. Thus, the signal is transduced from another cell to the cellular receptors embedded in the cell membrane [117]; as a result, a response is triggered and invagination of the cell membrane begins, which leads to the formation of a globular structure inside the cell [118], which is released in the cytoplasm and forms early endosomes. The action of the trans-Golgi network (TGN) on early endosomes results in the formation of late endosomes containing essential cellular components, including proteins, nucleic acids, and their derivatives [119], and the maturation of these late endosomes containing intraluminal vesicles (ILVs) to multivesicular bodies (MVBs) occurs through the action of ESCRT [120]. From there, round, nano-sized exosomes, rich in proteins, secondary metabolites, nucleic acids, and their derivatives, are released from cells into the intercellular space. This release, driven by osmotic pressure from the vacuole, highlights a unique aspect of their biogenesis [121,122]. While exosome generation and release primarily occur from multivesicular bodies (MVBs), some are also released via TRPML1-mediated lysosomal exocytosis. Remarkably, defects in lysosome functionality may increase MVB fusion with the plasma membrane, leading to a surge in exosome production [123,124]. Figure 5 illustrates the proposed biogenesis model for exosomes from MVBs in a plant cell.

## 4. Methods for the Isolation of Exosomes

Although exosomes are produced within a cell and are naturally secreted out of the cell into the extracellular space, for use in the pharmaceutical industry, exosomes need to be isolated by different methods [125]. Additionally, different methods like ultracentrifugation, immunoaffinity, size-based isolation (ultrafiltration, size-exclusion chromatography (SEC), flow field-flow fractionation), and precipitation are being used for the isolation and extraction of exosomes from their source [126]. Selecting an appropriate isolation method for exosome isolation is very important. As exosomes from a single source were isolated by using different approaches, their characteristics were comparatively divergent based on the isolation method being adopted [127]. Likewise, Ginseng-derived exosomes have also shown variance in terms of physicochemical characteristics after being isolated by using different techniques, including ultracentrifugation, EXOQuick, and a combination of EXO-Quick and ultracentrifugation [53]. In addition to physicochemical characteristics, the quality and quantity of exosomes are also altered depending on the nature of the method adopted for exosome isolation [128]. This clearly indicates that the characteristics of exosomes depend on the isolation techniques. Therefore, some insights into applied isolation methods are discussed herein, and the applications of these isolation methods in applied research for the isolation of plant-derived exosomes are reported in Table 2.

### 4.1. Ultracentrifugation Method

Ultracentrifugation is considered to be an effective method for exosome isolation. It is relatively costly and time-consuming, but the extracted amount of extracellular vesicles is higher when compared to other procedures used for the isolation of exosomes [50]. The ultracentrifugation method is mainly adopted by scientists who demand good purity of extracellular vesicles for proteomic and clinical research [24]. Because ultracentrifugation is confined to the extraction of particles of identical size and density rather than exosomes, there is a possibility of contamination [131]. Sucrose gradient separation (SGS) ultracentrifugation is a powerful technique, and is an improvement of the traditional ultracentrifugation method for the isolation of exosomes. The exosomes isolated from this method are of high purity [57]. Along with higher purity, the (SGS) method is considered to be cost-effective in terms of a high yield of exosomes [51].

### 4.2. Immunoaffinity

The immunoaffinity method of exosome isolation is based on the principle of immunoaffinity capturing. In this method, the exosomes are isolated by the marking of particular protein components present within extracellular vesicles from a supernatant [132]. Through the use of the immunoaffinity method, exosomes with retained morphological characteristics are isolated, resulting in the extraction of high-quality specific-type exosomes [133]. This method is cost-effective for specific types of exosome isolation within a short time, and because of this, it is frequently used for the diagnosis of different diseases by capturing exosome biomarkers [134]. Immunoaffinity capturing is a convenient method for the isolation of exosomes, but its limitation is that only the exosomes with specific targeted proteins can be captured, whereas the exosomes that lack targeted proteins remain in the supernatant [135]. Subsequently, the use of this method is limited, mainly due to the low availability of specific biomarkers for exosome capturing and isolation [44].

### 4.3. Size-Exclusion Chromatography (SEC)

The use of size exclusion chromatography for the isolation of extracellular vesicles (EVs) in clinical and therapeutic research is increasing because by adopting this procedure, the structural and morphological integrity of EVs is retained [136]. Size exclusion chromatography is an efficient method for isolating extracellular vesicles in proteomic research [137]. Furthermore, comparative studies have revealed its credibility regarding time consumption and quality retention; because of these features, this method is preferred by scientists [138]. In another study, a comparison is drawn between the SEC isolation method and the differential centrifugation (DC) method, and it has been recommended that the SEC isolation technique performs better than DC for research on extracellular vesicles in downstream studies [139]. Similarly, in the comparison study for EV isolation techniques, SEC, and ultracentrifugation, it has been determined that SEC is substantially superior for isolating highly functional EVs [140]. Moreover, the SEC comprises only a single-step isolation of exosomes/Es [141]. Hence, this isolation method is considered to be better than other isolation methods as the physicochemical properties of exosomes are retained after isolation, giving good quality exosomes, and it is also a cost-effective procedure, but isolated exosomes are quantitatively fewer in comparison to other conventionally used isolation methods [142]. In addition to this, advancements are being made in the SEC approach. Recently, the SEC, along with immobilized metal affinity chromatography, has been used to isolate exosomes, and isolated exosomes are highly pure. Additionally, the method is termed Fast Performance Liquid Chromatography (FPLC) [143].

### 4.4. Ultrafiltration

Ultrafiltration is based on the isolation of exosomes concerning size, The procedure is carried out in specially designed ultrafiltration tubes or units [144]. In a study, the ultrafiltration method with slight modification was compared with the conventional ultracentrifugation method, and it was reported that exosomes isolated by the use of ultrafiltration are of better quality and give a greater quantity of exosomes with better biotic stability [145]. To improve the concentration and quality of isolated exosomes, ultrafiltration (UF) in combination with other approaches, especially size exclusion chromatography (SEC), is used for the isolation of exosomes [146]. The coupling of ultrafiltration with SEC is a good technique for the isolation of EVs, and it has been noticed that this combination reduces the chances of contamination, and the highly pure EVs can be isolated with ease [147]. Therefore, it is highly recommended to use both of these techniques together for the isolation of extracellular vesicles for compositional and functional studies [148]. Similarly, in another study, the coupling of ultrafiltration centrifugation was considered as a facilitation tool for therapeutic and clinical research regarding exosome isolation, as it ensures the structural stability of isolated exosomes [149].

### 4.5. Flow Field-Flow Fractionation

Asymmetric-flow field-flow fractionation (AF4) technology is becoming more prevalent in pharmaceutical research to prepare nano-medicines [150]. AF4 is considered an efficient method for separation, and it is better used in drug production by ensuring the compositional, structural, and morphological stability of nano-medicines [151]. In this procedure, different types of field-flow fractionations, such as gravitational field and electric field, are applied in a forward and reverse manner within a device with a flat thin laminar flow channel with a permeable membrane at the bottom line to the source sample, and due to pressure exerted by the field ‘exosomes’ are isolated through downward movement from the permeable membrane at the bottom [152]. This AF4 method seems to be a time-saving technique, as the isolation of exosomes from the given sample takes much less time than that of other methods, and by adopting this method, the uniformity of particle size is also maintained [153]. In addition to its enormous advantages, it has some drawbacks, such as that it can only be used to extract exosomes from small samples, and particles in the sample should be the same size because particles with different morphology cannot be screened separately [154].

### 4.6. Precipitation

Exosome isolation by the precipitation method is carried out by using a precipitant material. The precipitation method is widely acknowledged as a cost-effective and convenient strategy because the isolation procedure is accomplished without the need for specialized equipment [149]. In a comparative study between ultracentrifugation and precipitation by the use of the ExoQuick method, the exosomes were isolated from blood plasma cells, and the adopted precipitation isolation method was proven to be six times faster than ultracentrifugation [155]. In another comparative study on the efficiency of exosome isolation methods, the ExoQuick method is declared as a better performer in terms of exosome isolation efficiency, when compared to both ultracentrifugation and exoEasy methods [156]. Furthermore, cold acetone has also been used as a precipitant for the isolation of extracellular vesicles via a precipitation method known as the protein organic solvent precipitation method (PROSPR), and it has been reported that high-purity EVs are generated during the process. In addition to this, it can be used effectively in clinical applications [157]. Another precipitation technique for the isolation of EVs, called aqueous two-phase system (ATPS), has been used for the extraction of extracellular vesicles from different organic sources, including plant lysate, and also used for EV isolation [158]. In addition, plant-derived exosomes can be isolated by varying percentages of polyethylene glycol (PEG), even though the production is relatively lower than that of the ultracentrifugation method [50].

### 4.7. Other Methods of Isolation

There are several other methods of isolation that are not commonly used at a commercial scale. This is because, on the basis of the aptamer in microfluidics, the microchannels have been used to isolate exosomes. This procedure is termed aptamer-based exosome isolation microfluidics, and the method is considered good for a low sample size [159].While the isolation of exosomes by the application of periodic negative pressure oscillations on an anodic aluminum oxide membrane coupled with air pressure within the EXODUS device is also being suggested, the reported procedure is termed EXODUS (exosome detection via the ultrafast-isolation system). In comparison to other isolation methods, EXODUS is a quick procedure with relatively high yield and high purity [160]. Moreover, the capillary-channeled polymer (C-CP) fiber spin-down tip method is also considered an efficient, less time-consuming, and relatively cheap method for the isolation of PDENs from different kinds of samples [161].

Along with advancements in isolation methods, techniques to increase exosome biosynthesis are also being explored. Recently, as a pretreatment for exosome isolation, to increase exosome biosynthesis for added yield, the plant cell wall was degraded by the action of digestive enzymes, which increased the production and release of exosomes [96]. With the advances in exosome research, new isolation techniques for exosomes are being reported, and similarly, new methods are being introduced after some improvements to existing techniques. As a result, these contributions have enabled the use of exosomes in therapeutics, as discussed herein.

## 5. Therapeutic Importance of Exosomes

Exosomes contribute to various cell processes, including apoptosis, angiogenesis, antigen presentation, cellular proliferation and differentiation, receptor-mediated endocytosis, cell signaling, and inflammation [162]. At present, exosomes are also commonly used in biomedical applications because of their source-specific characteristics, which enable their usage as biomarkers [163]. So far, their competency as biomarkers of various noninfectious diseases is playing a positive role in therapeutics [164]. They are being used as biomarkers in cardiovascular disorders [165], biomarkers for many types of malignant and non-malignant tumors [166,167,168,169], and biomarkers for disorders related to the brain [170,171,172]. Harnessing exosomes’ potential for disease diagnosis in therapeutics is not its limitation; they are even used for pathophysiological processes [173]. As in the case of infectious diseases, exosomes play a bi-dimensional role as transmitters of pathogens, causing more infections, and also trigger an immune response in the host, and inhibit the spread of infections [174]. Consequently, they are proven to be effective as a treatment agent for chronic inflammatory diseases [175]. Additionally, in certain cases, they can act as modulators for restricted growth, as in a condition known as androgenetic alopecia (AGA), which is related to hair loss in humans, where exosomes positively influence hair growth by molecular signaling to hair follicles, resulting in hair restoration [176]. However, the therapeutic potential of virus-associated exosomes is quoted as a mediator of immune responses in the body against viral infections [30].

Plant-derived exosomes are in the spotlight of scientific research because of their great therapeutic importance, which is acknowledged globally, and because they can be used as a therapeutic agent for various disease treatments [49]. This unmatchable potential in therapeutics is because of the edibility of plant-derived exosomes, which can be consumed orally for the treatment of diseases [63]. Additionally, plant-derived exosomes display minimal cytotoxicity for human cells by averting oxidative stress [97]. Therefore, their safe cytotoxic profile has enabled their potential use even in cancer treatment [177].

Plant-derived exosomes, sourced from various plants like aloe, lemon, ginger, turmeric, grapes, and strawberries, reveal a significant therapeutic effect. These nanovesicles can serve as both bio-components and drug delivery agents for treating conditions such as tumors and neurological disorders [178]. As a drug delivery agent, exosomes facilitate the delivery of proteins, siRNAs, DNA, and expression vectors by cargo loading and exosome engineering [179]. Similarly, exosomes derived from plants have great potential to act as a curative in digestion, and can regulate the immune system while inducing modulations in gut microbiota [180]. This fact is further proven by ginseng-derived exosomes’ potential to act as a curative in colitis progression by inhibiting inflammatory cytokines [54]. Furthermore, isolated plant exosomes hold therapeutic importance for treating lung disorders and even combating virus-borne diseases like COVID-19. Notably, ginger-derived exosomes have displayed anti-inflammatory effects and acted as an inhibitor for the attachment of *Porphyromonas gingivalis* to oral epithelial cells [181,182]. The diverse therapeutic potential of plant-derived exosomes, as supported by numerous studies, is further detailed in Table 2.

## 6. Plant-Derived Exosomes as a Targeted Drug Delivery Agent

Antibiotics and non-steroidal anti-inflammatory medications are frequently administered at high concentrations as part of traditional therapeutic approaches, with the objective of lowering inflammation [183]. Due to the fact that exosomes are naturally stable and extremely biocompatible for uptake by cells and tissues, they have the potential to function as innovative tools for targeted drug and gene delivery [184], whereas the adverse effects of conventional medications are unavoidable. In the past decades, plant exosomes have been reported to possess the capacity to be loaded with biological components intended for the treatment of various medical conditions [31]. A renowned anti-inflammatory drug, methotrexate (MTX), which is used to treat a number of diseases, also possesses cytotoxic side effects [185]. However, grapefruit exosomes tailored with MTX considerably reduced the cytotoxic effect of MTX with a considerable increase in its curative ability for easing dextran sulfate sodium (DSS)-induced colitis in animals [186]. Furthermore, ginger-derived nanovesicles fabricated with siRNA also result in a potential curative for ulcerative colitis [187]. So when plant-derived exosomes are loaded with nano drugs, they have increased pharmacological potential for usage as targeted drug delivery agents [188].

The sight-specific targeted application of plant-derived exosomes can inhibit cancer proliferation with no cytotoxic effect on normal cells [189]. Similarly, exosomes derived from citrus limon have proven the ability to target tumor sites and induce the tumor necrosis factor-related apoptosis-inducing ligand (TRAIL)-mediated apoptotic pathway, resulting in a regression of cancerous cells [190]. This illustrates that exosomes isolated from different plant sources can act as a nano-medicine in cancer therapy [191]. This is because of highly expressive miRNA content in plant-derived exosomes, which could lead to the regulation of cancer progression pathways [192]. Meanwhile, exosome engineering with different drugs is being carried out in order to boost their effectiveness for targeted cancer treatment with minimal side effects [193]. So, for this, exosomes are being altered for application in cancer therapy through a variety of strategies, including biological modifications, physical modifications, chemical modifications, and immunological modifications [194]. Meanwhile, for targeted delivery of these tailored nano-vesicles, chemotherapy and immunotherapy are being employed [195]. Furthermore, it has also been reported that exosomes derived from *Asparagus cochinchinensis* (Lour.) Merr. when coated with PEG exhibited enhanced anti-tumor response [177]. Likewise, in vivo studies have revealed that the targeted delivery of doxorubicin (DOX)-loaded ginger nanovesicles enhanced colon tumor regression [196]. Correspondingly, indocyanine green (ICG), when loaded on aloe-derived exosomes, leads to melanoma regression when targeted to melanoma cells [197].

In order to develop new strategies for the treatment of cancer, exosomes are being engineered to carry multiple cargos as a customized drug delivery agent [198]. As in the case of aloe exosomes engineered with dual drugs (DOX) and (ICG), the system exhibited high tumor regression capacity and is proposed as a targeted curative for breast cancer treatment [199]. Moreover, Astaxanthin (AST) is considered a potential anticancer drug [200], but with limited stability [201]. However, when exosomes derived from broccoli were engineered with poly (lactic-co-glycolic acid) (PLGA) and AST nanocomposites, this led to better stability and increased anticancer efficiency [202].

Similarly, plant-derived exosomes are being customized for the targeted delivery of proteins such as heat shock protein 70 (HSP70) while being utilized as agents for prospective medications for cancer treatments [61]. Grapefruit-derived exosomes have proven their potential as a targeted carrier of HSP70 and bovine serum albumin (BSA) to colon cancer cells and human peripheral blood mononuclear cells [109]. In the case of nucleic acids, plant-derived exosomes modified with miR-18a increased the inhibition of liver metastasis [203]. On the other hand, folic acid-modified exosomes have been employed to achieve targeted siRNA distribution in order to stimulate tumor regression [204]. Likewise, exosomes derived from kiwi fruit have been customized for siRNA-induced targeted gene delivery [205]. In a similar case, exosomes from broccoli were altered for exogenous miRNA delivery, resulting in the induction of toxicity in Caco-2 cells [206]. These findings signify the role of plant-derived exosomes as targeted drug delivery agents. However, numerous strategies are being employed to induce the fabrication of these nano-vesicles for utilization as targeted deliverers [207]. Here, we have presented several strategies that are frequently used for tailoring plant-derived exosomes.

### 6.1. Drug-Loading Methods for Targeted Treatments

With biocompatibility and exceptional drug loading efficiency, plant-derived exosomes have appeared as efficient drug delivery agents for targeted disease treatments [95]. The tailoring of exosomes with biological components is essential for the targeted treatment of diseases [208]. Generally, methods like sonication, extrusion, incubation and electroporation [209], transfection [210], and freeze-thawing [211] are used for the tailoring of plant-derived exosomes.

#### 6.1.1. Incubation

Incubation is a relatively cost-effective and easy method for tailoring exosomes with desired drugs without affecting the structural integrity of the exosomes [20]. In this approach, exosomes are tailored by incubation with desired nano-drugs to acquire the exosome composite [206]. Additionally, plant-derived exosomes tailored by adopting the incubation method also show high stability while being used in therapeutic approaches [197].

#### 6.1.2. Extrusion

The extrusion method involves enabling drug loading by exosome modification through extrusion [212]. This can be carried out by centrifugal force [213], a mini extruder like polycarbonate membranes [214], or by microfluidic fabrication [215]. It is reliable, as it presents higher drug loading ability with size uniformity [184], but it can result in changing the characterization of exosomes [216]. This method is being adopted for the treatment of multiple medical implications by engineered exosomes [217].

#### 6.1.3. Sonication

The sonication technique involves mixing the nanoparticle to be loaded along with exosomes in solution, followed by sonicating [218]. By adopting the sonication method, drug loading ability is enhanced; however, this results in structural damage due to the intense mechanical force induced by high-frequency ultrasound [219]. Meanwhile, disrupted membrane integrity can be improved through repair, which can be achieved by incubation at 37 °C for one hour [220].

#### 6.1.4. Transfection

Transfection involves delivering foreign nucleic acids to the targeted nano-vesicles [221], and there are several approaches to transfection, including viral-induced transfection and chemical-induced transfection [222]. Through this approach, the cost-effective loading of amino acids and nucleic acids in exosomes is enabled, carrying higher stability [34]. Accordingly, this approach is extremely effective for gene therapy by using RNA and DNA-loaded exosomes [223].

#### 6.1.5. Electroporation

Electroporation involves the introduction of small molecules into membranous vesicles through increasing membrane permeability by means of exposure to short electric impulses [224]. It is an exceedingly efficient approach to deliver functional genes for gene therapies [225] and for loading chemotherapeutic nano-medicines in exosomes for targeted chemotherapy [226]. Subsequently, electroporation encourages the addition of hydrophilic small molecules with exosomes, along with an enhancement of the incorporation of nucleic acids into exosomes [227]. On the other hand, with the adoption of electroporation for cargo loading, the aggregation of functional cargos is unavoidable due to strong electric fields causing temporary membrane permeabilization [184].

#### 6.1.6. Freeze Thawing

The freeze-thawing technique involves adding the desired cargo with exosomes at room temperature, and later subjecting them to an extremely low temperature, followed by thawing at room temperature and procedural repetition [228]. This approach can be aimed at drug loading for targeted cancer therapies by membrane fabrication [229]. However, by adopting this approach, because subsequent protein deactivation can happen [20], exosome aggregation, size variation, and minimal cargo loading are also its outcomes [230].

**Figure 6:**
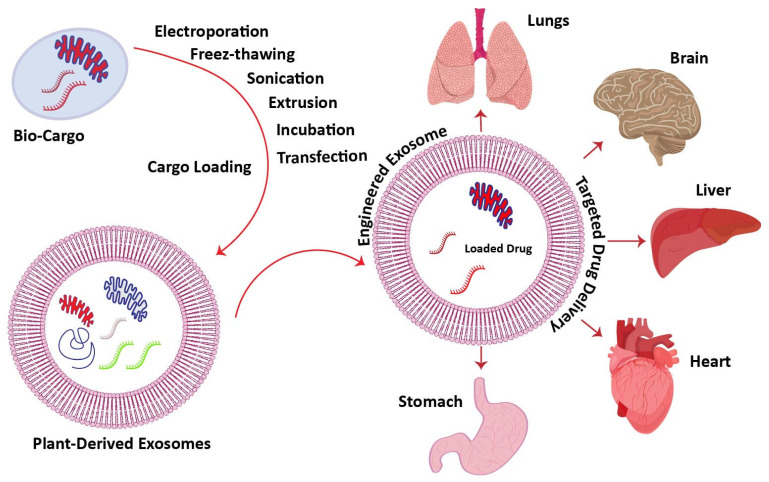
Exosome engineering with targeted drugs by drug-loading methods for targeted therapies.

With these drug loading approaches, the use of exosomes as a targeted drug delivery vessel has presented their competence as persuaders of cross-kingdom regulations through inter-kingdom communications. This factor is further stated herein, whereas exosome engineering for targeted therapies is presented in Figure 6.

## 7. Plant-Derived Exosomes as Cross-Kingdom Regulators

Being biological carriers, the extracellular vesicles have the capacity to induce inter-kingdom communication, which mediates immune responses [231]. EVs are responsible for transmitting communication from one cell to another, and they contain a substantial amount of miRNAs [232]. The RNA cargos in extracellular vesicles have significant benefits in research due to their capacity to regulate gene expression in the recipient cell, leading to cross-kingdom communication, affecting antigen presentation, immunological stimulation and suppression, and the transmission of virulence agents, which all contribute to the spread or inhibition of an infection [233]. Extracellular vesicles contain miRNAs and noncoding regulatory RNAs, which serve as a significant driver of inter-kingdom bidirectional communication [234,235]. The concept of cross-kingdom regulation, which suggests that dietary plant miRNAs influence human gene expression, remains highly controversial. A recent study, however, identified 350 circulating plant miRNAs in human plasma, hinting at their dietary absorption. Furthermore, these pmiRNAs were predicted to primarily target pathways involved in neurogenesis and nervous system development, opening new avenues for research and potentially supporting plant-induced cross-kingdom regulation [236].

This emerging understanding of cross-kingdom communication is particularly relevant as the administration of plant-derived exosomes for treating ailments is becoming more prevalent, with exosomal miRNAs from edible plants showing promise in inducing cross-kingdom regulatory mechanisms for treating diseases like SARS-CoV-2 by targeting the viral transcriptome [104,237]. In a similar way, citrus nanovesicles also contain many essential protein bio-cargos [238] and the biochemical and bioinformatic compositions reflect citrus-derived exosomes’ abilities to interact with their surroundings [239], and through these exosomes, the proliferation of cancer cells is halted in humans [106].

Recently, the effect of lemon juice nanovesicles (LNVs) has been observed in human dermal fibroblasts and zebrafish embryos, resulting in a reduction in reactive oxygen species (ROS). This effect is thought to be a result of the activation of the AhR/Nrf2 signaling pathway after being influenced by treatment with LNVs [240]. Similarly, exosomes from mulberry regulate the AhR-COPS8-mediated anti-inflammatory pathway, leading to the effective treatment of numerous inflammatory conditions in mammals [235].

Meanwhile, Parkinson’s disease and myocardial infarction can be treated with carrot-derived exosomes that are equipped with high antioxidant capabilities, with a tendency for cellular uptake [111]. Furthermore, it is detected that blueberry-derived exosomes induce modulation in inflammatory gene expression after uptake by the human stabilized endothelial cell line (EA.hy926) [113]. Similarly, exosomes derived from *Portulaca oleracea* L. led to a reduction in Zbtb7b expression level; as a result, double-positive CD4+CD8+ T cells were formed from CD4+ T cells [62]. Likewise, broccoli-derived exosomes induced regulations in mammalian dendritic cells, causing colitis inhibition through the activation of adenosine monophosphate (AMP)-activated protein kinase [241].

Exosomes derived from *Momordica charantia* L. downregulated the levels of IL-1β, IL-6, and TNF-α and upregulated the IL-10 level in mammals, and this suggests their use as a potential curative for Ulcerative colitis [242]. As a matter of fact, Momordica’s exosomes exhibit a highly expressed miR5813, and they also mediated p-AKT/AKT and p-PI3K/PI3K levels, enabling anti-glioma usage [243].

In MC3T3-E1 cells, plum-derived exosomes regulated the expression of osteoblastic transcription factors, and they also raised levels of p38, JNK, phosphorylated BMP-2, and Smad1 proteins. Moreover, they downregulated TRAP-positive cells in osteoclasts. This is an indication of the effectiveness of plum-derived exosomes in the treatment of bone disorders [244].

**Figure 7:**
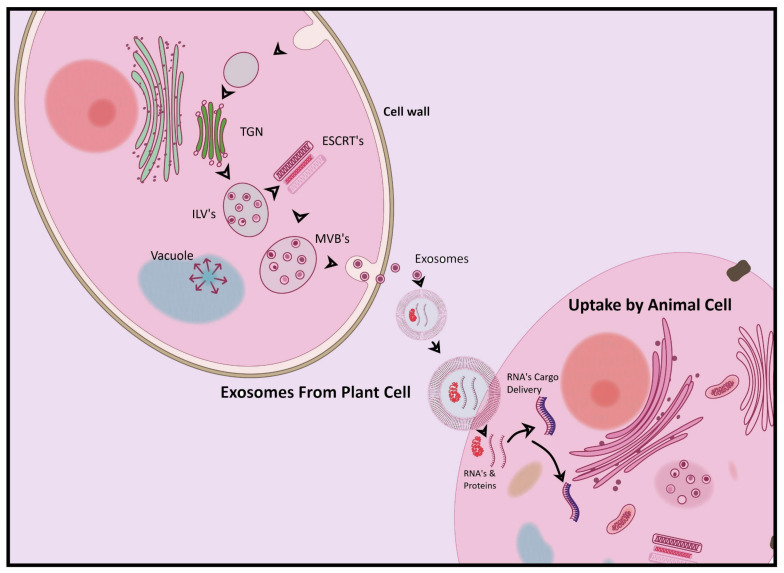
Modulation of cross-kingdom regulation by plant-derived exosomes.

All these results demonstrate that plant-derived exosomes act as cross-kingdom communicators, leading to cross-kingdom regulation. However, the precise mechanism by which these exosomes are taken up by mammalian cells opens up critical avenues for further scientific research. Despite this, plant-derived exosomes exhibit the ability to communicate across kingdoms and hold significant potential for treating a variety of human diseases. The modulation of cross-kingdom regulation by the cross-kingdom communication of plant-derived exosomes is represented in Figure 7.

## 8. Conclusions

Plant-derived exosomes are increasingly recognized as important cross-kingdom communicators with significant therapeutic potential, though research in this area is still in its early stages. These naturally occurring nanovesicles show promise as both nanodrugs and biological nanocarriers, building on the historical use of plants in herbal medicine. While effective isolation and characterization are crucial for their research utility, scientists are actively developing new and improved techniques, and advanced studies have already started to reveal their therapeutic potential. Despite their growing use in pharmaceuticals and therapeutics, a universal standard for characterizing plant-derived exosomes is still lacking. As isolation and characterization methods continue to evolve with new scientific insights, ongoing research into plant-derived exosomes is vital for advancing disease detection and treatment. In conclusion, the vast diversity of the plant kingdom means that each species produces unique exosomes with distinct biological compositions and therapeutic capabilities, offering opportunities for future research into their diverse applications, which will ultimately lead to the provision of potent plant-based therapeutics exhibiting potential for inducing cross-kingdom regulations, consequently treating human ailments.

## Figures and Tables

**Table 2:** Plant-derived exosomes’ therapeutic potential and adopted Isolation methods.

Plant Source	Exosome Source	Isolation Method (IM)	Therapeutic Potential	Targeted Disease	Cargo Loaded	Bioactivity Validated in	Reference
*Artemisia annua* L.	Herbaceous plant parts	Sucrose gradient separation	Inter-kingdom communication, tumor regression	Cancer	None	Mice/Cells	[33]
Cica (*Cantella asiatica*)	Leaves	Ultracentrifugation aqueous two-phase system (ATPS)	Cosmeceutical product	Skin health/aging	None	Cells	[48]
Purslane (*Portulaca oleracea*)	Leaves	Ultracentrifugation	Cosmeceutical product	Skin health/aging	None	Cells	[48]
Green tea (*Camellia sinensis*)	Leaves	Ultracentrifugation aqueous two-phase system (ATPS)	Cosmeceutical product	Skin health/aging	None	Cells	[48]
Ginseng (*P. ginseng*)	Roots	Ultracentrifugation Aqueous two-phase systems (ATPS)	Cosmeceutical product	Skin health/aging	None	Cells	[48]
Ginger (*Zingiber officinalis*)	Rhizome Roots	Sucrose gradient separation	Prospective protective agent against alcohol induced live injury	Alcohol-induced liver damage	None	Mice/Cells	[71]
	Rhizome	Sucrose gradient separation	Effective for the treatment and prevention of colitis-associated cancer and inflammatory bowel disease	Inflammatory bowel disease and colitis-associated cancer	None	Mice/Cells	[72]
	Peeled Hawaiian ginger roots	Sucrose gradient separation	Treatment of viral infections like COVID-19	Lung inflammation	None	Mice/Cells	[102]
	Rhizome var. Gajah	Ultracentrifugation and precipitation (Polyethylene glycol 6000) (PEG-6000)	Potential drug delivery agent and potential nano-nutrient carrier	Not specified	None	Cells	[103]
	Fresh Rhizome	PEG precipitation	miRNA capacity for targeting transcriptome of SARS-CoV-2	SARS-CoV-2	mi-RNA	None	[104]
Garlic (*Allium sativum* L.)	Bulbs	PEG precipitation Ultracentrifugation	Regulation of 6-phosphofructo-2-kinase/fructose-2, 6-biphosphatase 3 (PFKFB3) expression for inhibition of inflammatory response in mice	Nonalcoholic fatty liver disease	None	Mice/Cells	[55]
	Bulbs	Microfiltration followed by PEG precipitation, then ultracentrifugation, followed by microfiltration	Regulation of (PFKFB3) expression for mediation of glucose metabolic reprogramming leading to attenuation of inflammatory responses	Chronic Inflammation	None	Mice/Cells	[56]
Curcumae Rhizoma (*Curcuma longa* L.)	Rhizome	Sucrose gradient separation	Potential nano carrier for Astragalus components to enhance anti-tumor activity	Cancer	*Astragalus* components (AC)	Mice/Cells	[57]
Tartary buckwheat (*Fagopyrum tataricum*)	Seeds	Sucrose gradient separation	Prospective natural ingredients for the regulation of postprandial glucose	Not specified	None	None	[59]
Dandelion (*Taraxacum officinale*)	Herbaceous Part	Ultracentrifugation	Effective for the reduction in intermittent hypoxia-induced hypertension	Hypoxia-induced hypertension	None	Mice	[60]
Tomato (*Solanum lycopersicum*)	Fruit	Ultracentrifugation	Potential drug delivery agent	Not specified	None	Cells	[61]
Grapefruit (*Citrus paradise*)	Fruit	Ultracentrifugation	Potential drug delivery agent	Not specified	None	Cells	[61]
	Fruit	Ultracentrifugation	Potential carrier of proteins to human cells	Not specified	Proteins	Mice/Cells	[109]
	Edible portion of fruit	PEG Precipitation	miRNA capacity for targeting the transcriptome of SARS-CoV-2	SARS-CoV-2	mi-RNA	None	[104]
	Fruit Juice	Ultracentrifugation	Inhibition of tumor proliferation	Cancer	None	Cells	[106]
Turmeric (*Curcuma longa*)	Rhizome	Sucrose gradient separation	Colitis treatment	Ulcerative colitis	None	Mice/Cells	[63]
*Salvia dominica*	Hairy roots	Ultracentrifugation	Prospective antitumor agent	Not specified	None	Cells	[93]
*Morinda officinalis*	Roots	Ultracentrifugation	Drug carriers and therapeutic agents	Not specified	None	Mice/Cells	[96]
Strawberry (*Fragaria x ananassa*)	Fruits	Ultracentrifugation	Potential drug carrier	Not specified	None	Cells	[97]
Apple	Fruit (Fuji apples)	Ultracentrifugation	mRNA expression modulation of intestinal transporters	Human epithelial colorectal adenocarcinoma	None	Cells	[98]
	Fruit (Golden Delicious) (*Malus domestica* sp.)	Ultracentrifugation	Induce an anti-inflammatory effect in primary dermal fibroblasts	Skin aging	None	Cells	[99]
	Fruit (Sun Fuji) (*Mallus pumila*)	Ultracentrifugation	Regulation of mRNA expression of intestinal transport materials	Not specified	None	Cells	[100]
	Fruit (Sun Fuji) (*Malus pumila*)	Ultracentrifugation	mRNA expression regulation of intestinal transporters	Not specified	None	Cells	[101]
	Fruit (Golden delicious) (*Malus domestica* sp.)	Ultracentrifugation	Anti-inflammatory effect	Inflammation	None	Cells	[129]
Quina plant (*Cinchona ledgeriana*)	Friable Callus	Ultracentrifugation and Precipitation (Polyethylene glycol 6000) (PEG-6000)	Potential drug delivery agent and potential nano-nutrient carrier	Not specified	None	Cells	[103]
Citrus (*Citrus reticulate*)	Fruit Juice	Ultracentrifugation, followed by sucrose gradient centrifugation	Inhibition of citrus blue mold on citrus fruit	Citrus blue mold caused by *Penicillium italicum* (plant disease)	None	Fungus in vitro	[105]
Sweet orange (*C. sinensis*)	Fruit Juice	Ultracentrifugation	Inhibition of tumor proliferation	Cancer	None	Cells	[106]
Lemon (*C. limon*)	Fruit Juice	Ultracentrifugation	Inhibition of tumor proliferation	Cancer	None	Cells	[106]
Bitter orange (*C. aurantium*)	Fruit Juice	Ultracentrifugation	Inhibition of tumor proliferation	Cancer	None	Cells	[106]
Golden Cherry (*Physalis minima*)	Fruits	PEG precipitation	Treatment of photoaging	Anti-photoaging	None	None	[107]
Yam (*Dioscorea japonica*)	Tuber (Fresh Juice)	Sucrose gradient separation	Stimulation of osteoblasts formation in mice leading to prevention of osteoporosis	Osteoporosis	None	Mice/Cells	[108]
*Flos Sophorae Immaturus* (*Sophora japonica* L.)	Flowers	Ultracentrifugation	Promotion of spinal cord repair by regulation of oxidative stress in microenvironment, prospectively use for CNS diseases treatment	Spinal cord injury	None	Mice/Cells	[130]
Tobacco (*Nicotiana tabacum*)	Callus culture and BY-2 suspension culture	Ultracentrifugation and Precipitation	Potential carrier for cellular uptake	Not specified	None	Cells	[110]
Carrot (*Daucus carota* subsp. *Sativus*)	Fresh Juice of Edible Taproot	Ultrafiltration followed by size exclusion chromatography	Possible curative for Parkinson’s disease and myocardial infarction	Parkinson’s disease and myocardial infarction	None	Cells	[111]
Blueberry	Fruits (Apoplastic Fluid)	Ultracentrifugation	Immunomodulatory therapies	Not specified	None	Cells	[112]

For isolation methods, differential centrifugation has been carried out for the removal of large particles before the execution of the quoted methods.

## Data Availability

Not applicable.

## References

[1] Christenhusz M., Byng J. (2016). The number of known plant species in the world and its annual increase. Phytotaxa.

[2] Ahmad T. (2013). Shahabuddin. The uses of medicinal plants in the treatment of diseases. Eur. Acad. Res..

[3] Bhuiyan F.R., Howlader S., Raihan T., Hasan M. (2020). Plants metabolites: Possibility of natural therapeutics against the COVID-19 pandemic. Front. Med..

[4] Saminathan A., Zajac M., Anees P., Krishnan Y. (2022). Organelle-level precision with next-generation targeting technologies. Nat. Rev. Mater..

[5] Mathivanan S., Ji H., Simpson R.J. (2010). Exosomes: Extracellular organelles important in intercellular communication. J. Proteom..

[6] Pegtel D.M., Gould S.J. (2019). Exosomes. Annu. Rev. Biochem..

[7] Fox A.S., Yoon S.B. (1970). DNA-induced transformation in Drosophila: Locus-specificity and the establishment of transformed stocks. Proc. Natl. Acad. Sci. USA.

[8] Harding C., Heuser J., Stahl P. (1983). Receptor-mediated endocytosis of transferrin and recycling of the transferrin receptor in rat reticulocytes. J. Cell Biol..

[9] Pan B.T., Johnstone R.M. (1983). Fate of the transferrin receptor during maturation of sheep reticulocytes in vitro: Selective externalization of the receptor. Cell.

[10] Johnstone R.M. (2006). Exosomes biological significance: A concise review. Blood Cells Mol. Dis..

[11] Ahn S.H., Ryu S.W., Choi H., You S., Park J., Choi C. (2022). Manufacturing therapeutic exosomes: From bench to industry. Mol. Cells.

[12] Théry C., Zitvogel L., Amigorena S. (2002). Exosomes: Composition, biogenesis and function. Nat. Rev. Immunol..

[13] Chernyshev V.S., Rachamadugu R., Tseng Y.H., Belnap D.M., Jia Y., Branch K.J., Butterfield A.E., Pease L.F., Bernard P.S., Skliar M. (2015). Size and shape characterization of hydrated and desiccated exosomes. Anal. Bioanal. Chem..

[14] Johnstone R.M., Adam M., Hammond J.R., Orr L., Turbide C. (1987). Vesicle formation during reticulocyte maturation. Association of plasma membrane activities with released vesicles (exosomes). J. Biol. Chem..

[15] Subha D., Harshnii K., Madhikiruba K.G., Nandhini M., Tamilselvi K.S. (2023). Plant derived exosome-like nanovesicles: An updated overview. Plant Nano Biol..

[16] An Q., van Bel A.J.E., Hückelhoven R. (2007). Do plant cells secrete exosomes derived from multivesicular bodies?. Plant Signal. Behav..

[17] van Niel G., D’Angelo G., Raposo G. (2018). Shedding light on the cell biology of extracellular vesicles. Nat. Rev. Mol. Cell Biol..

[18] Ou X., Wang H., Tie H., Liao J., Luo Y., Huang W., Yu R., Song L., Zhu J. (2023). Novel plant-derived exosome-like nanovesicles from Catharanthus roseus: Preparation, characterization, and immunostimulatory effect via TNF-α/NF-κB/PU.1 axis. J. Nanobiotechnol..

[19] Yamashita T., Takahashi Y., Takakura Y. (2018). Possibility of exosome-based therapeutics and challenges in production of exosomes eligible for therapeutic application. Biol. Pharm. Bull..

[20] Kimiz-Gebologlu I., Oncel S.S. (2022). Exosomes: Large-scale production, isolation, drug loading efficiency, and biodistribution and uptake. J. Control. Release.

[21] Mahmoudi M. (2021). The need for robust characterization of nanomaterials for nanomedicine applications. Nat. Commun..

[22] Kowal J., Tkach M., Théry C. (2014). Biogenesis and secretion of exosomes. Curr. Opin. Cell Biol..

[23] Alzhrani G.N., Alanazi S.T., Alsharif S.Y., Albalawi A.M., Alsharif A.A., Abdel-Maksoud M.S., Elsherbiny N. (2021). Exosomes: Isolation, characterization, and biomedical applications. Cell Biol. Int..

[24] Lai J.J., Chau Z.L., Chen S.Y., Hill J.J., Korpany K.V., Liang N.W., Lin L.H., Lin Y.H., Liu J.K., Liu Y.C. (2022). Exosome processing and characterization approaches for research and technology development. Adv. Sci..

[25] Zebrowska A., Skowronek A., Wojakowska A., Widlak P., Pietrowska M. (2019). Metabolome of exosomes: Focus on vesicles released by cancer cells and present in human body fluids. Int. J. Mol. Sci..

[26] Kugeratski F.G., Hodge K., Lilla S., McAndrews K.M., Zhou X., Hwang R.F., Zanivan S., Kalluri R. (2021). Quantitative proteomics identifies the core proteome of exosomes with syntenin-1 as the highest abundant protein and a putative universal biomarker. Nat. Cell Biol..

[27] Rutter B.D., Innes R.W. (2017). Extracellular vesicles isolated from the leaf apoplast carry stress-response proteins. Plant Physiol..

[28] Elkommos-Zakhary M., Rajesh N., Beljanski V. (2022). Exosome RNA sequencing as a tool in the search for cancer biomarkers. Non-Coding RNA.

[29] Zhao Z., Yu S., Li M., Gui X., Li P. (2018). Isolation of exosome-like nanoparticles and analysis of microRNAs derived from coconut water based on small RNA high-throughput sequencing. J. Agric. Food Chem..

[30] Peng Y., Yang Y., Li Y., Shi T., Luan Y., Yin C. (2023). Exosome and virus infection. Front. Immunol..

[31] Cai Y., Zhang L., Zhang Y., Lu R. (2022). Plant-derived exosomes as a drug-delivery approach for the treatment of inflammatory bowel disease and colitis-associated cancer. Pharmaceutics.

[32] Zhao X., Wu D., Ma X., Wang J., Hou W., Zhang W. (2020). Exosomes as drug carriers for cancer therapy and challenges regarding exosome uptake. Biomed. Pharmacother..

[33] Liu J., Xiang J., Jin C., Ye L., Wang L., Gao Y., Lv N., Zhang J., You F., Qiao H. (2023). Medicinal plant-derived mtDNA via nanovesicles induces the cGAS-STING pathway to remold tumor-associated macrophages for tumor regression. J. Nanobiotechnol..

[34] Fu S., Wang Y., Xia X., Zheng J.C. (2020). Exosome engineering: Current progress in cargo loading and targeted delivery. NanoImpact.

[35] Song Y., Kim Y., Ha S., Sheller-Miller J., Yoo J., Choi C., Park C.H. (2021). The emerging role of exosomes as novel therapeutics: Biology, technologies, clinical applications, and the next. Am. J. Reprod. Immunol..

[36] Mu N., Li J., Zeng L. (2023). Plant-derived exosome-like nanovesicles: Current progress and prospects. Int. J. Nanomed..

[37] Xu X.-H., Yuan T.-J., Dad H.A., Shi M.-Y., Huang Y.-Y., Jiang Z.-H., Peng L.-H. (2021). Plant exosomes as novel nanoplatforms for microRNA transfer stimulate neural differentiation of stem cells in vitro and in vivo. Nano Lett..

[38] Chuo S.T.-Y., Chien J.C.-Y., Lai C.P.-K. (2018). Imaging extracellular vesicles: Current and emerging methods. J. Biomed. Sci..

[39] Lim J., Choi M., Lee H., Kim Y.-H., Han J.-Y., Lee E.S., Cho Y. (2019). Direct isolation and characterization of circulating exosomes from biological samples using magnetic nanowires. J. Nanobiotechnol..

[40] Kowal J., Arras G., Colombo M., Jouve M., Morath J.P., Primdal-Bengtson B., Dingli F., Loew D. (2016). Proteomic comparison defines novel markers to characterize heterogeneous populations of extracellular vesicle subtypes. Proc. Natl. Acad. Sci. USA.

[41] He C., Zheng S., Luo Y., Wang B. (2018). Exosome theranostics: Biology and translational medicine. Theranostics.

[42] Cocozza F., Grisard E., Martin-Jaular L., Mathieu M., Théry C. (2020). SnapShot: Extracellular vesicles. Cell.

[43] Zhu L., Sun H.-T., Wang S., Huang S.-L., Zheng Y., Wang C.-Q., Hu B.-Y., Qin W., Zou T.-T., Fu Y. (2020). Isolation and characterization of exosomes for cancer research. J. Hematol. Oncol..

[44] Greening D.W., Xu R., Ji H., Tauro B.J., Simpson R.J. (2015). A protocol for exosome isolation and characterization: Evaluation of ultracentrifugation, density-gradient separation, and immunoaffinity capture methods. Methods Mol. Biol..

[45] Li M., Li S., Du C., Zhang Y., Li Y., Chu L., Han X., Galons H., Zhang Y., Sun H. (2020). Exosomes from different cells: Characteristics, modifications, and therapeutic applications. Eur. J. Med. Chem..

[46] Li D., Luo H., Ruan H., Chen Z., Chen S., Wang B., Xie Y. (2021). Isolation and identification of exosomes from feline plasma, urine and adipose-derived mesenchymal stem cells. BMC Vet. Res..

[47] Mahdipour E. (2022). *Beta vulgaris* juice contains biologically active exosome-like nanoparticles. Tissue Cell.

[48] Cho J.H., Hong Y.D., Kim D., Park S.J., Kim J.S., Kim H.-M., Yoon E.J., Cho J.-S. (2022). Confirmation of plant-derived exosomes as bioactive substances for skin application through comparative analysis of keratinocyte transcriptome. Appl. Biol. Chem..

[49] Kim J., Li S., Zhang S., Wang J. (2022). Plant-derived exosome-like nanoparticles and their therapeutic activities. Asian J. Pharm. Sci..

[50] Kalarikkal S.P., Prasad D., Kasiappan R., Chaudhari S.R., Sundaram G.M. (2020). A cost-effective polyethylene glycol-based method for the isolation of functional edible nanoparticles from ginger rhizomes. Sci. Rep..

[51] Kim J., Lee Y.-H., Wang J., Kim Y.K., Kwon I.K. (2022). Isolation and characterization of ginseng-derived exosome-like nanoparticles with sucrose cushioning followed by ultracentrifugation. SN Appl. Sci..

[52] Cao M., Yan H., Han X., Weng L., Wei Q., Sun X., Lu W., Wei Q., Ye J., Cai X. (2019). Ginseng-derived nanoparticles alter macrophage polarization to inhibit melanoma growth. J. Immunother. Cancer.

[53] Jang J., Jeong H., Jang E., Kim E., Yoon Y., Jang S., Jeong H.-S., Jang G. (2023). Isolation of high-purity and high-stability exosomes from ginseng. Front. Plant Sci..

[54] Kim J., Zhang S., Zhu Y., Wang R., Wang J. (2023). Amelioration of colitis progression by ginseng-derived exosome-like nanoparticles through suppression of inflammatory cytokines. J. Ginseng Res..

[55] Liu J., Li W., Bian Y., Jiang X., Zhu F., Yin F., Yin L., Song X., Guo H., Liu J. (2023). Garlic-derived exosomes regulate PFKFB3 expression to relieve liver dysfunction in high-fat diet-fed mice via macrophage-hepatocyte crosstalk. Phytomedicine.

[56] Bian Y., Li W., Jiang X., Yin F., Yin L., Zhang Y., Guo H., Liu J. (2023). Garlic-derived exosomes carrying miR-396e shapes macrophage metabolic reprograming to mitigate the inflammatory response in obese adipose tissue. J. Nutr. Biochem..

[57] Yang X., Peng Y., Wang Y.-e., Zheng Y., He Y., Pan J., Liu N., Xu Y., Ma R., Zhai J. (2023). Curcumae Rhizoma Exosomes-like nanoparticles loaded Astragalus components improve the absorption and enhance anti-tumor effect. J. Drug Deliv. Sci. Technol..

[58] You J.Y., Kang S.J., Rhee W.J. (2021). Isolation of cabbage exosome-like nanovesicles and investigation of their biological activities in human cells. Bioact. Mater..

[59] Li D., Cao G., Yao X., Yang Y., Yang D., Liu N., Yuan Y., Nishinari K., Yang X. (2023). Tartary buckwheat-derived exosome-like nanovesicles against starch digestion and their interaction mechanism. Food Hydrocoll..

[60] Zhang X., Pan Z., Wang Y., Liu P., Hu K. (2023). Taraxacum officinale-derived exosome-like nanovesicles modulate gut metabolites to prevent intermittent hypoxia-induced hypertension. Biomed. Pharmacother..

[61] Kilasoniya A., Garaeva L., Shtam T., Spitsyna A., Putevich E., Moreno-Chamba B., Salazar-Bermeo J., Komarova E., Malek A., Valero M. (2023). Potential of plant exosome vesicles from Grapefruit (Citrus × paradisi) and Tomato (*Solanum lycopersicum*) juices as functional ingredients and targeted drug delivery vehicles. Antioxidants.

[62] Zhu M.Z., Xu H.M., Liang Y.J., Xu J., Yue N.N., Zhang Y., Tian C.M., Yao J., Wang L.S., Nie Y.Q. (2023). Edible exosome-like nanoparticles from Portulaca oleracea L mitigate DSS-induced colitis via facilitating double-positive CD4^+^CD8^+^T cells expansion. J. Nanobiotechnol..

[63] Liu C., Yan X., Zhang Y., Yang M., Ma Y., Zhang Y., Xu Q., Tu K., Zhang M. (2022). Oral administration of turmeric-derived exosome-like nanovesicles with anti-inflammatory and pro-resolving bioactions for murine colitis therapy. J. Nanobiotechnol..

[64] Zhu Q., Huang L., Yang Q., Ao Z., Yang R., Krzesniak J., Lou D., Hu L., Dai X., Guo F. (2021). Metabolomic analysis of exosomal-markers in esophageal squamous cell carcinoma. Nanoscale.

[65] Wang X., Tian L., Lu J., Ng I.O.-L. (2022). Exosomes and cancer—Diagnostic and prognostic biomarkers and therapeutic vehicle. Oncogenesis.

[66] Patil S.M., Sawant S.S., Kunda N.K. (2020). Exosomes as drug delivery systems: A brief overview and progress update. Eur. J. Pharm. Biopharm..

[67] Öztürk K., Kaplan M., Çalış S. (2024). Effects of nanoparticle size, shape, and zeta potential on drug delivery. Int. J. Pharm..

[68] Rasmussen M.K., Pedersen J.N., Marie R. (2020). Size and surface charge characterization of nanoparticles with a salt gradient. Nat. Commun..

[69] Midekessa G., Godakumara K., Ord J., Viil J., Lättekivi F., Dissanayake K., Kopanchuk S., Rinken A., Andronowska A., Bhattacharjee S. (2020). Zeta potential of extracellular vesicles: Toward understanding the attributes that determine colloidal stability. ACS Omega.

[70] Teng Y., He J., Zhong Q., Zhang Y., Lu Z., Guan T., Pan Y., Luo X., Feng W., Ou C. (2022). Grape exosome-like nanoparticles: A potential therapeutic strategy for vascular calcification. Front. Pharmacol..

[71] Zhuang X., Deng Z.B., Mu J., Zhang L., Yan J., Miller D., Feng W., McClain C.J., Zhang H.G. (2015). Ginger-derived nanoparticles protect against alcohol-induced liver damage. J. Extracell. Vesicles.

[72] Zhang M., Viennois E., Prasad M., Zhang Y., Wang L., Zhang Z., Han M.K., Xiao B., Xu C., Srinivasan S. (2016). Edible ginger-derived nanoparticles: A novel therapeutic approach for the prevention and treatment of inflammatory bowel disease and colitis-associated cancer. Biomaterials.

[73] Kamble S., Agrawal S., Cherumukkil S., Sharma V., Jasra R.V., Munshi P. (2022). Revisiting zeta potential, the key feature of interfacial phenomena, with applications and recent advancements. ChemistrySelect.

[74] Panigrahi A.R., Srinivas L., Panda J. (2022). Exosomes: Insights and therapeutic applications in cancer. Transl. Oncol..

[75] Skotland T., Sandvig K., Llorente A. (2017). Lipids in exosomes: Current knowledge and the way forward. Prog. Lipid Res..

[76] Haraszti R.A., Didiot M.C., Sapp E., Leszyk J., Shaffer S.A., Rockwell H.E., Gao F., Narain N.R., DiFiglia M., Kiebish M.A. (2016). High-resolution proteomic and lipidomic analysis of exosomes and microvesicles from different cell sources. J. Extracell. Vesicles.

[77] Lam S.M., Zhang C., Wang Z., Ni Z., Zhang S., Yang S., Huang X., Mo L., Li J., Lee B. (2021). A multi-omics investigation of the composition and function of extracellular vesicles along the temporal trajectory of COVID-19. Nat. Metab..

[78] Peterka O., Jirásko R., Chocholoušková M., Kuchař L., Wolrab D., Hájek R., Vrána D., Strouhal O., Melichar B., Holčapek M. (2020). Lipidomic characterization of exosomes isolated from human plasma using various mass spectrometry techniques. Biochim. Biophys. Acta (BBA) Mol. Cell Biol. Lipids.

[79] Skotland T., Hessvik N.P., Sandvig K., Llorente A. (2019). Exosomal lipid composition and the role of ether lipids and phosphoinositides in exosome biology. J. Lipid Res..

[80] Wang Y.T., Shi T., Srivastava S., Kagan J., Liu T., Rodland K.D. (2020). Proteomic analysis of exosomes for discovery of protein biomarkers for prostate and bladder cancer. Cancers.

[81] Gurung S., Perocheau D., Touramanidou L., Baruteau J. (2021). The exosome journey: From biogenesis to uptake and intracellular signalling. Cell Commun. Signal..

[82] Liu X., Locasale J.W. (2017). Metabolomics: A primer. Trends Biochem. Sci..

[83] Du Y., Dong J.-H., Chen L., Liu H., Zheng G.-E., Chen G.-Y., Cheng Y. (2022). Metabolomic identification of serum exosome-derived biomarkers for bipolar disorder. Oxidative Med. Cell. Longev..

[84] Ye M., Wang J., Pan S., Zheng L., Wang Z.-W., Zhu X. (2022). Nucleic acids and proteins carried by exosomes of different origins as potential biomarkers for gynecologic cancers. Mol. Ther. Oncolytics.

[85] Dai Y., Cao Y., Köhler J., Lu A., Xu S., Wang H. (2021). Unbiased RNA-Seq-driven identification and validation of reference genes for quantitative RT-PCR analyses of pooled cancer exosomes. BMC Genom..

[86] Zhang Y., Bi J., Huang J., Tang Y., Du S., Li P. (2020). Exosome: A review of its classification, isolation techniques, storage, diagnostic and targeted therapy applications. Int. J. Nanomed..

[87] Zhang L., Yu D. (2019). Exosomes in cancer development, metastasis, and immunity. Biochim. Biophys. Acta Rev. Cancer.

[88] Zhang Z.G., Buller B., Chopp M. (2019). Exosomes—Beyond stem cells for restorative therapy in stroke and neurological injury. Nat. Rev. Neurol..

[89] Jiao Y., Xu P., Shi H., Chen D., Shi H. (2021). Advances on liver cell-derived exosomes in liver diseases. J. Cell. Mol. Med..

[90] Danesh A., Inglis H.C., Jackman R.P., Wu S., Deng X., Muench M.O., Heitman J.W., Norris P.J. (2014). Exosomes from red blood cell units bind to monocytes and induce proinflammatory cytokines, boosting T-cell responses in vitro. Blood.

[91] Gao M., Gao W., Papadimitriou J.M., Zhang C., Gao J., Zheng M. (2018). Exosomes—The enigmatic regulators of bone homeostasis. Bone Res..

[92] Urzì O., Raimondo S., Alessandro R. (2021). Extracellular vesicles from plants: Current knowledge and open questions. Int. J. Mol. Sci..

[93] Boccia E., Alfieri M., Belvedere R., Santoro V., Colella M., Del Gaudio P., Moros M., Dal Piaz F., Petrella A., Leone A. (2022). Plant hairy roots for the production of extracellular vesicles with antitumor bioactivity. Commun. Biol..

[94] Chukhchin D.G., Bolotova K., Sinelnikov I., Churilov D., Novozhilov E. (2019). Exosomes in the phloem and xylem of woody plants. Planta.

[95] Di Raimo R., Mizzoni D. (2023). Oral treatment with plant-derived exosomes restores redox balance in H_2_O_2_-treated mice. Antioxidants.

[96] Zhao Q., Liu G., Liu F., Xie M., Zou Y., Wang S., Guo Z., Dong J., Ye J., Cao Y. (2023). An enzyme-based system for extraction of small extracellular vesicles from plants. Sci. Rep..

[97] Perut F., Roncuzzi L., Avnet S. (2021). Strawberry-derived exosome-like nanoparticles prevent oxidative stress in human mesenchymal stromal cells. Biomolecules.

[98] Fujita D., Arai T., Komori H., Shirasaki Y., Wakayama T., Nakanishi T., Tamai I. (2018). Apple-derived nanoparticles modulate expression of Organic-Anion-Transporting Polypeptide (OATP) 2B1 in Caco-2 cells. Mol. Pharm..

[99] Trentini M., Zanolla I., Zanotti F., Tiengo E., Licastro D., Dal Monego S., Lovatti L., Zavan B. (2022). Apple derived exosomes improve collagen type I production and decrease MMPs during aging of the skin through downregulation of the NF-κB pathway as mode of action. Cells.

[100] Arai M., Komori H., Fujita D., Tamai I. (2021). Uptake pathway of apple-derived nanoparticle by intestinal cells to deliver its cargo. Pharm. Res..

[101] Komori H., Fujita D., Shirasaki Y., Zhu Q., Iwamoto Y., Nakanishi T., Nakajima M., Tamai I. (2021). MicroRNAs in apple-derived nanoparticles modulate intestinal expression of Organic Anion-Transporting Peptide 2B1/SLCO2B1 in Caco-2 cells. Drug Metab. Dispos..

[102] Teng Y., Xu F., Zhang X., Mu J., Sayed M., Hu X., Lei C., Sriwastva M.S., Kumar A., Sundaram K. (2021). Plant-derived exosomal microRNAs inhibit lung inflammation induced by exosomes SARS-CoV-2 Nsp12. Mol. Ther..

[103] Ratnadewi D., Widjaja C.H., Barlian A., Amsar R.M., Ana I.D., Hidajah A.C., Notobroto H.B., Wungu T.D.K. (2022). Isolation of native plant-derived exosome-like nanoparticles and their uptake by human cells. HAYATI J. Biosci..

[104] Kalarikkal S.P., Sundaram G.M. (2021). Edible plant-derived exosomal microRNAs: Exploiting a cross-kingdom regulatory mechanism for targeting SARS-CoV-2. Toxicol. Appl. Pharmacol..

[105] Yin C., Zhu H., Lao Y., Jiang Y., Gong L. (2023). MicroRNAs in the exosome-like nanoparticles from orange juice inhibit Citrus blue mold caused by Penicillium italicum. LWT—Food Sci. Technol..

[106] Stanly C., Alfieri M., Ambrosone A. (2020). Grapefruit-derived micro and nanovesicles show distinct metabolome profiles and anticancer activities in the A375 human melanoma cell line. Cells.

[107] Setiadi E.V., Adlia A., Barlian A., Ayuningtyas D.F., Rachmawati H. (2024). Development and characterization of a gel formulation containing golden cherry exosomes (Physalis minima) as a potential anti-photoaging. Pharm. Nanotechnol..

[108] Hwang J.-H., Park Y.-S., Kim H.-S., Kim D.-H., Lee S.-H., Lee C.-H., Lee S.-H., Kim J.-E., Lee S., Kim H.M. (2023). Yam-derived exosome-like nanovesicles stimulate osteoblast formation and prevent osteoporosis in mice. J. Control. Release.

[109] Garaeva L., Kamyshinsky R., Kil Y., Varfolomeeva E., Verlov N., Komarova E., Garmay Y., Landa S., Burdakov V., Myasnikov A. (2021). Delivery of functional exogenous proteins by plant-derived vesicles to human cells in vitro. Sci. Rep..

[110] Kocholata M., Prusova M., Malinska H.A., Maly J., Janouskova O. (2022). Comparison of two isolation methods of tobacco-derived extracellular vesicles, their characterization and uptake by plant and rat cells. Sci. Rep..

[111] Kim D.K., Rhee W.J. (2021). Antioxidative effects of carrot-derived nanovesicles in cardiomyoblast and neuroblastoma cells. Pharmaceutics.

[112] Nguyen T.N.-G., Pham C.V., Chowdhury R., Patel S., Jaysawal S.K., Hou Y., Xu H., Jia L., Duan A., Tran P.H.-L. (2023). Development of blueberry-derived extracellular nanovesicles for immunomodulatory therapy. Pharmaceutics.

[113] De Robertis M., Sarra A., D’Oria V., Mura F., Bordi F., Postorino P. (2020). Blueberry-Derived Exosome-Like Nanoparticles Counter the Response to TNF-α-Induced Change on Gene Expression in EA.hy926 Cells. Biomolecules.

[114] Hansen L.L., Nielsen M.E. (2017). Plant exosomes: Using an unconventional exit to prevent pathogen entry?. J. Exp. Bot..

[115] Wu B., Liu D.A., Guan L., Myint P.K., Chin L., Dang H., Xu Y., Ren J., Li T., Yu Z. (2023). Stiff matrix induces exosome secretion to promote tumour growth. Nat. Cell Biol..

[116] Dawson T.R., Weaver A.M. (2023). Niche tension controls exosome production. Nat. Cell Biol..

[117] Liu J., Shapiro J.I. (2003). Endocytosis and signal transduction: Basic science update. Biol. Res. Nurs..

[118] Cui Y., Shen J., Gao C., Zhuang X., Wang J., Jiang L. (2016). Biogenesis of plant prevacuolar multivesicular bodies. Mol. Plant.

[119] Han Q.F., Li W.J., Hu K.S., Gao J., Zhai W.L., Yang J.H., Zhang S.J. (2022). Exosome biogenesis: Machinery, regulation, and therapeutic implications in cancer. Mol. Cancer.

[120] Li X., Bao H., Wang Z., Wang M., Fan B., Zhu C., Chen Z. (2018). Biogenesis and function of multivesicular bodies in plant immunity. Front. Plant Sci..

[121] Krylova S.V., Feng D. (2023). The machinery of exosomes: Biogenesis, release, and uptake. Int. J. Mol. Sci..

[122] Dmitrieff S., Nédélec F. (2015). Membrane mechanics of endocytosis in cells with turgor. PLoS Comput. Biol..

[123] Kim M.S., Muallem S., Kim S.H., Kwon K.B., Kim M.S. (2019). Exosomal release through TRPML1-mediated lysosomal exocytosis is required for adipogenesis. Biochem. Biophys. Res. Commun..

[124] Adams S.D., Csere J., D’angelo G., Carter E.P., Romao M., Arnandis T., Dodel M., Kocher H.M., Grose R., Raposo G. (2021). Centrosome amplification mediates small extracellular vesicle secretion via lysosome disruption. Curr. Biol..

[125] Wu X., Showiheen S.A.A., Sun A.R., Crawford R., Xiao Y., Mao X., Prasadam I. (2019). Exosomes Extraction and Identification. Methods Mol. Biol..

[126] Suharta S., Barlian A., Hidajah A.C., Notobroto H.B., Ana I.D., Indariani S., Wungu T.D.K., Wijaya C.H. (2021). Plant-derived exosome-like nanoparticles: A concise review on its extraction methods, content, bioactivities, and potential as functional food ingredient. J. Food Sci..

[127] Dash M., Palaniyandi K., Ramalingam S., Sahabudeen S., Raja N.S. (2021). Exosomes isolated from two different cell lines using three different isolation techniques show variation in physical and molecular characteristics. Biochim. Biophys. Acta Biomembr..

[128] Xu W.-M., Li A., Chen J.-J., Sun E.-J. (2023). Research development on exosome separation technology. J. Membr. Biol..

[129] Trentini M., Zanotti F., Tiengo E., Camponogara F., Degasperi M., Licastro D., Lovatti L., Zavan B. (2022). An apple a day keeps the doctor away: Potential role of miRNA 146 on macrophages treated with exosomes derived from apples. Biomedicines.

[130] Chen J., Wu J., Mu J., Li L., Hu J., Lin H., Cao J., Gao J. (2023). An antioxidative sophora exosome-encapsulated hydrogel promotes spinal cord repair by regulating oxidative stress microenvironment. Nanomedicine.

[131] Chen J., Li P., Zhang T., Xu Z., Huang X., Wang R., Du L. (2021). Review on strategies and technologies for exosome isolation and purification. Front. Bioeng. Biotechnol..

[132] Sharma P., Ludwig S., Muller L., Hong C.S., Kirkwood J.M., Ferrone S., Whiteside T.L. (2018). Immunoaffinity-based isolation of melanoma cell-derived exosomes from plasma of patients with melanoma. J. Extracell. Vesicles.

[133] Yousif G., Qadri S., Parray A., Akhthar N., Shuaib A., Haik Y. (2022). Exosomes derived neuronal markers: Immunoaffinity isolation and characterization. Neuromol. Med..

[134] Sharafeldin M., Yan S., Jiang C., Tofaris G.K., Davis J.J. (2023). Alternating magnetic field-promoted nanoparticle mixing: The on-chip immunocapture of serum neuronal exosomes for Parkinson’s disease diagnostics. Anal. Chem..

[135] Jang Y.O., Ahn H.S., Dao T.N.T., Hong J., Shin W., Lim Y.M., Chung S.J., Lee J.H., Liu H., Koo B. (2023). Magnetic transferrin nanoparticles (MTNs) assay as a novel isolation approach for exosomal biomarkers in neurological diseases. Biomater. Res..

[136] Monguió-Tortajada M., Gálvez-Montón C., Bayes-Genis A., Roura S., Borràs F.E. (2019). Extracellular vesicle isolation methods: Rising impact of size-exclusion chromatography. Cell. Mol. Life Sci..

[137] Lane R.E., Korbie D., Trau M., Hill M.M. (2019). Optimizing size exclusion chromatography for extracellular vesicle enrichment and proteomic analysis from clinically relevant samples. Proteomics.

[138] Gámez-Valero A., Monguió-Tortajada M., Carreras-Planella L., Franquesa M., Beyer K., Borràs F.E. (2016). Size-exclusion chromatography-based isolation minimally alters extracellular vesicles’ characteristics compared to precipitating agents. Sci. Rep..

[139] Davis C.N., Phillips H., Tomes J.J., Swain M.T., Wilkinson T.J., Brophy P.M., Morphew R.M. (2019). The importance of extracellular vesicle purification for downstream analysis: A comparison of differential centrifugation and size exclusion chromatography for helminth pathogens. PLoS Neglected Trop. Dis..

[140] Mol E.A., Goumans M.J., Doevendans P.A., Sluijter J.P.G., Vader P. (2017). Higher functionality of extracellular vesicles isolated using size-exclusion chromatography compared to ultracentrifugation. Nanomedicine.

[141] Böing A.N., van der Pol E., Grootemaat A.E., Coumans F.A., Sturk A., Nieuwland R. (2014). Single-step isolation of extracellular vesicles by size-exclusion chromatography. J. Extracell. Vesicles.

[142] Sidhom K., Obi P.O., Saleem A. (2020). A review of exosomal isolation methods: Is size exclusion chromatography the best option?. Int. J. Mol. Sci..

[143] Bellotti C., Lang K., Kuplennik N., Sosnik A., Steinfeld R. (2021). High-grade extracellular vesicles preparation by combined size-exclusion and affinity chromatography. Sci. Rep..

[144] Wang W., Lian J.Q., Wang P.Z., Pan L., Ji X.Y., Bai X.F., Jia Z.S. (2007). Isolation of exosomes derived from dendritic cells by ultrafiltration centrifugalization and their morphologic characteristics. Xi Bao Yu Fen Zi Mian Yi Xue Za Zhi Chin. J. Cell. Mol. Immunol..

[145] He L., Zhu D., Wang J., Wu X. (2019). A highly efficient method for isolating urinary exosomes. Int. J. Mol. Med..

[146] Shu S., Allen C.L., Benjamin-Davalos S., Koroleva M., MacFarland D., Minderman H., Ernstoff M.S. (2021). A rapid exosome isolation using ultrafiltration and size exclusion chromatography (REIUS) method for exosome isolation from melanoma cell lines. Methods Mol. Biol..

[147] Guerreiro E.M., Vestad B., Steffensen L.A., Aass H.C.D., Saeed M., Øvstebø R., Costea D.E., Galtung H.K., Søland T.M. (2018). Efficient extracellular vesicle isolation by combining cell media modifications, ultrafiltration, and size-exclusion chromatography. PLoS ONE.

[148] Benedikter B.J., Bouwman F.G., Vajen T., Heinzmann A.C.A., Grauls G., Mariman E.C., Wouters E.F.M., Savelkoul P.H., Lopez-Iglesias C., Koenen R.R. (2017). Ultrafiltration combined with size exclusion chromatography efficiently isolates extracellular vesicles from cell culture media for compositional and functional studies. Sci. Rep..

[149] Li P., Kaslan M., Lee S.H., Yao J., Gao Z. (2017). Progress in Exosome Isolation Techniques. Theranostics.

[150] Wagner M., Holzschuh S., Traeger A., Fahr A., Schubert U.S. (2014). Asymmetric flow field-flow fractionation in the field of nanomedicine. Anal. Chem..

[151] Bian J., Gobalasingham N., Purchel A., Lin J. (2023). The power of field-flow fractionation in characterization of nanoparticles in drug delivery. Molecules.

[152] Zhang H., Lyden D. (2019). Asymmetric-flow field-flow fractionation technology for exomere and small extracellular vesicle separation and characterization. Nat. Protoc..

[153] Wahlund K.G. (2013). Flow field-flow fractionation: Critical overview. J. Chromatogr. A.

[154] Gao J., Li A., Hu J., Feng L., Liu L., Shen Z. (2022). Recent developments in isolating methods for exosomes. Front. Bioeng. Biotechnol..

[155] Coughlan C., Bruce K.D., Burgy O., Boyd T.D., Michel C.R., Garcia-Perez J.E., Adame V., Anton P., Bettcher B.M., Chial H.J. (2020). Exosome isolation by ultracentrifugation and precipitation and techniques for downstream analyses. Curr. Protoc. Cell Biol..

[156] Jung H.H., Kim J.Y., Lim J.E., Im Y.H. (2020). Cytokine profiling in serum-derived exosomes isolated by different methods. Sci. Rep..

[157] Gallart-Palau X., Serra A., Wong A.S., Sandin S., Lai M.K., Chen C.P., Kon O.L., Sze S.K. (2015). Extracellular vesicles are rapidly purified from human plasma by PRotein Organic Solvent PRecipitation (PROSPR). Sci. Rep..

[158] Kırbaş O.K., Bozkurt B.T., Asutay A.B., Mat B., Ozdemir B., Öztürkoğlu D., Ölmez H., İşlek Z., Şahin F., Taşlı P.N. (2019). Optimized isolation of extracellular vesicles from various organic sources using aqueous two-phase system. Sci. Rep..

[159] Zhou Z., Chen Y., Qian X. (2022). Target-specific exosome isolation through aptamer-based microfluidics. Biosensors.

[160] Chen Y., Zhu Q., Cheng L., Wang Y., Li M., Yang Q., Hu L., Lou D., Li J., Dong X. (2021). Exosome detection via the ultrafast-isolation system: EXODUS. Nat. Methods.

[161] Jackson K.K., Mata C., Marcus R.K. (2023). A rapid capillary-channeled polymer (C-CP) fiber spin-down tip approach for the isolation of plant-derived extracellular vesicles (PDEVs) from 20 common fruit and vegetable sources. Talanta.

[162] Dyball L.E., Smales C.M. (2022). Exosomes: Biogenesis, targeting, characterization and their potential as “Plug & Play” vaccine platforms. Biotechnol. J..

[163] Gurunathan S., Kang M.H., Kim J.H. (2021). A comprehensive review on factors influences biogenesis, functions, therapeutic and clinical implications of exosomes. Int. J. Nanomed..

[164] Shivji G.G., Dhar R., Devi A. (2022). Role of exosomes and its emerging therapeutic applications in the pathophysiology of non-infectious diseases. Biomarkers.

[165] de Freitas R.C.C., Hirata R.D.C., Hirata M.H., Aikawa E. (2021). Circulating extracellular vesicles as biomarkers and drug delivery vehicles in cardiovascular diseases. Biomolecules.

[166] Rahbarghazi R., Jabbari N., Sani N.A., Asghari R., Salimi L., Kalashani S.A., Feghhi M., Etemadi T., Akbariazar E., Mahmoudi M. (2019). Tumor-derived extracellular vesicles: Reliable tools for Cancer diagnosis and clinical applications. Cell Commun. Signal..

[167] Chang W.H., Cerione R.A., Antonyak M.A. (2021). Extracellular vesicles and their roles in cancer progression. Methods Mol. Biol..

[168] Martellucci S., Orefice N.S., Angelucci A., Luce A., Caraglia M., Zappavigna S. (2020). Extracellular Vesicles: New Endogenous Shuttles for miRNAs in Cancer Diagnosis and Therapy?. Int. J. Mol. Sci..

[169] Salehi M., Sharifi M. (2018). Exosomal miRNAs as novel cancer biomarkers: Challenges and opportunities. J. Cell. Physiol..

[170] Dutta S., Hornung S., Taha H.B., Bitan G. (2023). Biomarkers for parkinsonian disorders in CNS-originating EVs: Promise and challenges. Acta Neuropathol..

[171] Hill A.F. (2019). Extracellular vesicles and neurodegenerative diseases. J. Neurosci..

[172] Hornung S., Dutta S., Bitan G. (2020). CNS-derived blood exosomes as a promising source of biomarkers: Opportunities and challenges. Front. Mol. Neurosci..

[173] Kavya A.N.V.L., Subramanian S., Ramakrishna S. (2022). Therapeutic applications of exosomes in various diseases: A review. Biomater. Adv..

[174] Rangel-Ramírez V.V., González-Sánchez H.M., Lucio-García C. (2023). Exosomes: From biology to immunotherapy in infectious diseases. Infect. Dis..

[175] Wang C., Xu M., Fan Q., Li C., Zhou X. (2023). Therapeutic potential of exosome-based personalized delivery platform in chronic inflammatory diseases. Asian J. Pharm. Sci..

[176] Gupta A.K., Hall D.C., Rapaport J.A., Paradise C.R. (2023). Exosomes and hair restoration. Adv. Cosmet. Surg..

[177] Zhang L., He F., Gao L., Cong M., Sun J., Xu J., Wang Y., Hu Y., Asghar S., Hu L. (2021). Engineering exosome-like nanovesicles derived from Asparagus cochinchinensis can inhibit the proliferation of hepatocellular carcinoma cells with better safety profile. Int. J. Nanomed..

[178] Barzin M., Bagheri A.M., Ohadi M., Abhaji A.M., Salarpour S., Dehghannoudeh G. (2023). Application of plant-derived exosome-like nanoparticles in drug delivery. Pharm. Dev. Technol..

[179] Dad H.A., Gu T.W., Zhu A.Q., Huang L.Q., Peng L.H. (2021). Plant exosome-like nanovesicles: Emerging therapeutics and drug delivery nanoplatforms. Mol. Ther..

[180] Yi Q., Xu Z., Thakur A., Zhang K., Liang Q., Liu Y., Yan Y. (2023). Current understanding of plant-derived exosome-like nanoparticles in regulating the inflammatory response and immune system microenvironment. Pharmacol. Res..

[181] Li A., Li D., Gu Y., Liu R., Tang X., Zhao Y., Qi F., Wei J., Liu J. (2023). Plant-derived nanovesicles: Further exploration of biomedical function and application potential. Acta Pharm. Sin. B.

[182] Sundaram K., Miller D.P., Kumar A., Teng Y., Sayed M., Mu J., Lei C., Sriwastva M.K., Zhang L., Yan J. (2019). Plant-derived exosomal nanoparticles inhibit pathogenicity of Porphyromonas gingivalis. iScience.

[183] Yang C., Merlin D. (2019). Nanoparticle-mediated drug delivery systems for the treatment of IBD: Current perspectives. Int. J. Nanomed..

[184] Chen H., Wang L., Zeng X., Schwarz H., Nanda H.S., Peng X., Zhou Y. (2021). Exosomes, a new star for targeted delivery. Front. Cell Dev. Biol..

[185] Hamed K.M., Dighriri I.M., Baomar A.F., Alharthy B.T., Alenazi F.E., Alali G.H., Alenazy R.H., Alhumaidi N.T., Alhulayfi D.H., Alotaibi Y.B. (2022). Overview of methotrexate toxicity: A comprehensive literature review. Cureus.

[186] Wang B., Zhuang X., Deng Z.B., Jiang H., Mu J., Wang Q., Xiang X., Guo H., Zhang L., Dryden G. (2014). Targeted drug delivery to intestinal macrophages by bioactive nanovesicles released from grapefruit. Mol. Ther..

[187] Zhang M., Wang X., Han M.K., Collins J.F., Merlin D. (2017). Oral administration of ginger-derived nanolipids loaded with siRNA as a novel approach for efficient siRNA drug delivery to treat ulcerative colitis. Nanomedicine.

[188] Chen X., Ji S., Yan Y., Lin S., He L., Huang X., Chang L., Zheng D. (2023). Engineered plant-derived nanovesicles facilitate tumor therapy: Natural bioactivity plus drug controlled release platform. Int. J. Nanomed..

[189] Kim K., Yoo H.J., Jung J.H., Lee R., Hyun J.K., Park J.H., Na D., Yeon J.H. (2020). Cytotoxic effects of plant sap-derived extracellular vesicles on various tumor cell types. J. Funct. Biomater..

[190] Raimondo S., Naselli F., Fontana S., Monteleone F., Lo Dico A., Saieva L., Zito G., Flugy A., Manno M., Di Bella M.A. (2015). Citrus limon-derived nanovesicles inhibit cancer cell proliferation and suppress CML xenograft growth by inducing TRAIL-mediated cell death. Oncotarget.

[191] Dhar R., Mukerjee N., Mukherjee D., Devi A., Jha S.K., Gorai S. (2024). Plant-derived exosomes: A new dimension in cancer therapy. Phytother. Res..

[192] Yin L., Yan L., Yu Q., Wang J., Liu C. (2022). Characterization of the MicroRNA profile of ginger exosome-like nanoparticles and their anti-Inflammatory effects in intestinal Caco-2 cells. J. Agric. Food Chem..

[193] Liu X., Xiao C., Xiao K. (2023). Engineered extracellular vesicles-like biomimetic nanoparticles as an emerging platform for targeted cancer therapy. J. Nanobiotechnol..

[194] Zhang M., Hu S., Liu L., Dang P., Liu Y., Sun Z., Qiao B., Wang C. (2023). Engineered exosomes from different sources for cancer-targeted therapy. Signal Transduct. Target. Ther..

[195] Chen Y., Wang L., Zheng M., Zhu C., Wang G., Xia Y., Blumenthal E.J., Mao W., Wan Y. (2022). Engineered extracellular vesicles for concurrent Anti-PDL1 immunotherapy and chemotherapy. Bioact. Mater..

[196] Zhang M., Xiao B., Wang H., Han M.K., Zhang Z., Viennois E., Xu C., Merlin D. (2016). Edible ginger-derived nano-lipids loaded with doxorubicin as a novel drug-delivery approach for colon cancer therapy. Mol. Ther..

[197] Zeng L., Wang H., Shi W., Chen L., Chen T., Chen G., Wang W., Lan J., Huang Z., Zhang J. (2021). Aloe derived nanovesicle as a functional carrier for indocyanine green encapsulation and phototherapy. J. Nanobiotechnol..

[198] Cheng Q., Shi X., Han M., Smbatyan G., Lenz H.J., Zhang Y. (2018). Reprogramming Exosomes as Nanoscale Controllers of Cellular Immunity. J. Am. Chem. Soc..

[199] Zeng L., Shi W., Wang H., Cheng X., Chen T., Wang L.L., Lan J., Sun W. (2022). Codelivery of π-π Stacked Dual Anticancer Drugs Based on Aloe-Derived Nanovesicles for Breast Cancer Therapy. ACS Appl. Mater. Interfaces.

[200] Faraone I., Sinisgalli C., Ostuni A., Armentano M.F., Carmosino M., Milella L., Russo D., Labanca F., Khan H. (2020). Astaxanthin anticancer effects are mediated through multiple molecular mechanisms: A systematic review. Pharmacol. Res..

[201] Martínez-Delgado A.A., Khandual S., Villanueva–Rodríguez S.J. (2017). Chemical stability of astaxanthin integrated into a food matrix: Effects of food processing and methods for preservation. Food Chem..

[202] Li C., Song Q., Yin X., Song R., Chen G. (2022). Preparation, characterization, and in vitro anticancer activity evaluation of broccoli-derived extracellular vesicle-coated astaxanthin nanoparticles. Molecules.

[203] Teng Y., Mu J., Hu X., Samykutty A., Zhuang X., Deng Z., Zhang L., Cao P., Yan J., Miller D. (2016). Grapefruit-derived nanovectors deliver miR-18a for treatment of liver metastasis of colon cancer by induction of M1 macrophages. Oncotarget.

[204] Li Z., Wang H., Yin H., Bennett C., Zhang H.G., Guo P. (2018). Arrowtail RNA for ligand display on ginger exosome-like nanovesicles to systemic deliver siRNA for cancer suppression. Sci. Rep..

[205] Huang H., Yi X., Wei Q., Li M., Cai X., Lv Y., Weng L., Mao Y., Fan W., Zhao M. (2023). Edible and cation-free kiwi fruit derived vesicles mediated EGFR-targeted siRNA delivery to inhibit multidrug resistant lung cancer. J. Nanobiotechnol..

[206] del Pozo-Acebo L., López de las Hazas M.-C., Tomé-Carneiro J., del Saz-Lara A., Gil-Zamorano J., Balaguer L., Chapado L.A., Busto R., Visioli F., Dávalos A. (2022). Therapeutic potential of broccoli-derived extracellular vesicles as nanocarriers of exogenous miRNAs. Pharmacol. Res..

[207] Herrmann I.K., Wood M.J.A., Fuhrmann G. (2021). Extracellular vesicles as a next-generation drug delivery platform. Nat. Nanotechnol..

[208] Raimondo S., Giavaresi G., Lorico A., Alessandro R. (2019). Extracellular vesicles as biological shuttles for targeted therapies. Int. J. Mol. Sci..

[209] Alzahrani F.A., Khan M.I. (2023). Plant-derived extracellular vesicles and their exciting potential as the future of next-generation drug delivery. Biomolecules.

[210] Jeong K., Yu Y.J., You J.Y., Rhee W.J., Kim J.A. (2020). Exosome-mediated microRNA-497 delivery for anti-cancer therapy in a microfluidic 3D lung cancer model. Lab A Chip.

[211] Haney M.J., Klyachko N.L., Zhao Y., Gupta R., Plotnikova E.G., He Z., Patel T., Piroyan A., Sokolsky M., Kabanov A.V. (2015). Exosomes as drug delivery vehicles for Parkinson’s disease therapy. J. Control. Release.

[212] Kenari A.N., Cheng L., Hill A.F. (2020). Methods for loading therapeutics into extracellular vesicles and generating extracellular vesicles mimetic-nanovesicles. Methods.

[213] Wan Y., Wang L., Zhu C., Zheng Q., Wang G., Tong J., Fang Y., Xia Y., Cheng G., He X. (2018). Aptamer-conjugated extracellular nanovesicles for targeted drug delivery. Cancer Res..

[214] Choo Y.W., Kang M., Kim H.Y., Han J., Kang S., Lee J.-R., Jeong G.-J., Kwon S.P., Song S.Y., Go S. (2018). M1 macrophage-derived nanovesicles potentiate the anticancer efficacy of immune checkpoint inhibitors. ACS Nano.

[215] Jo W., Jeong D., Kim J., Cho S., Jang S.C., Han C., Kang J.Y., Gho Y.S., Park J. (2014). Microfluidic fabrication of cell-derived nanovesicles as endogenous RNA carriers. Lab A Chip.

[216] Fuhrmann G., Serio A., Mazo M., Nair R., Stevens M.M. (2015). Active loading into extracellular vesicles significantly improves the cellular uptake and photodynamic effect of porphyrins. J. Control. Release.

[217] Xi X.M., Xia S.J., Lu R. (2021). Drug loading techniques for exosome-based drug delivery systems. Pharmazie.

[218] Kim M.S., Haney M.J., Zhao Y., Yuan D., Deygen I., Klyachko N.L., Kabanov A.V., Batrakova E.V. (2018). Engineering macrophage-derived exosomes for targeted paclitaxel delivery to pulmonary metastases: In vitro and in vivo evaluations. Nanomedicine.

[219] Danilushkina A.A., Emene C.C., Barlev N.A., Gomzikova M.O. (2023). Strategies for engineering of extracellular vesicles. Int. J. Mol. Sci..

[220] Kim M.S., Haney M.J., Zhao Y., Mahajan V., Deygen I., Klyachko N.L., Inskoe E., Piroyan A., Sokolsky M., Okolie O. (2016). Development of exosome-encapsulated paclitaxel to overcome MDR in cancer cells. Nanomedicine.

[221] Zhang Y., Liu Q., Zhang X., Huang H., Tang S., Chai Y., Xu Z., Li M., Chen X., Liu J. (2022). Recent advances in exosome-mediated nucleic acid delivery for cancer therapy. J. Nanobiotechnol..

[222] Chong Z.X., Yeap S.K. (2021). Transfection types, methods and strategies: A technical review. PeerJ.

[223] Munagala R., Aqil F., Jeyabalan J., Kandimalla R., Wallen M., Tyagi N., Wilcher S., Yan J., Schultz D.J., Spencer W. (2021). Exosome-mediated delivery of RNA and DNA for gene therapy. Cancer Lett..

[224] Shirley S.A., Heller R., Heller L.C., Lattime E.C., Gerson S.L. (2014). Chapter 7—Electroporation Gene Therapy. Gene Therapy of Cancer.

[225] Nikyar A., Bolhassani A. (2022). Electroporation: An effective method for in vivo gene delivery. Drug Deliv. Lett..

[226] Lennaárd A.J., Mamand D.R., Wiklander R.J., El Andaloussi S., Wiklander O.P.B. (2021). Optimised electroporation for loading of extracellular vesicles with doxorubicin. Pharmaceutics.

[227] Luan X., Sansanaphongpricha K., Myers I., Chen H., Yuan H., Sun D. (2017). Engineering exosomes as refined biological nanoplatforms for drug delivery. Acta Pharmacol. Sin..

[228] Sato Y.T., Umezaki K., Sawada S., Mukai S.-A., Sasaki Y., Harada N., Shiku H., Akiyoshi K. (2016). Engineering hybrid exosomes by membrane fusion with liposomes. Sci. Rep..

[229] Tran P.H.L., Wang T., Yin W., Tran T.T.D., Nguyen T.N.G., Lee B.J., Duan W. (2019). Aspirin-loaded nanoexosomes as cancer therapeutics. Int. J. Pharm..

[230] Ferreira D., Moreira J.N., Rodrigues L.R. (2022). New advances in exosome-based targeted drug delivery systems. Crit. Rev. Oncol. Hematol..

[231] Liu G., Kang G., Wang S., Huang Y., Cai Q. (2021). Extracellular vesicles: Emerging players in plant defense against pathogens. Front. Plant Sci..

[232] Urzì O., Gasparro R., Ganji N.R., Alessandro R. (2022). Plant-RNA in extracellular vesicles: The secret of cross-kingdom communication. Membranes.

[233] Munhoz da Rocha I.F., Amatuzzi R.F., Lucena A.C.R., Faoro H., Alves L.R. (2020). Cross-kingdom extracellular vesicles EV-RNA communication as a mechanism for host-pathogen interaction. Front. Cell. Infect. Microbiol..

[234] Stanton B.A. (2021). Extracellular vesicles and host–pathogen interactions: A review of inter-Kingdom signaling by small noncoding RNA. Genes.

[235] Sriwastva M.K., Deng Z.B., Wang B., Teng Y., Kumar A., Sundaram K., Mu J., Lei C., Dryden G.W., Xu F. (2022). Exosome-like nanoparticles from Mulberry bark prevent DSS-induced colitis via the AhR/COPS8 pathway. EMBO Rep..

[236] Olmi L., Pepe G., Helmer-Citterich M., Canini A., Gismondi A. (2023). Looking for plant microRNAs in human blood samples: Bioinformatics evidence and perspectives. Plant Foods Hum. Nutr..

[237] Xu Z., Xu Y., Zhang K., Liu Y., Liang Q., Thakur A., Liu W., Yan Y. (2023). Plant-derived extracellular vesicles (PDEVs) in nanomedicine for human disease and therapeutic modalities. J. Nanobiotechnol..

[238] Pocsfalvi G., Turiák L., Ambrosone A., del Gaudio P., Puska G., Fiume I., Silvestre T., Vékey K. (2018). Protein biocargo of citrus fruit-derived vesicles reveals heterogeneous transport and extracellular vesicle populations. J. Plant Physiol..

[239] Stanly C., Moubarak M., Fiume I., Turiák L. (2019). Membrane transporters in *Citrus clementina* fruit juice-derived nanovesicles. Int. J. Mol. Sci..

[240] Urzì O., Cafora M., Ganji N.R., Tinnirello V., Gasparro R., Raccosta S., Manno M., Corsale A.M., Conigliaro A., Pistocchi A. (2023). Lemon-derived nanovesicles achieve antioxidant and anti-inflammatory effects activating the AhR/Nrf2 signaling pathway. iScience.

[241] Deng Z., Rong Y., Teng Y., Mu J., Zhuang X., Tseng M., Samykutty A., Zhang L., Yan J., Miller D. (2017). Broccoli-derived nanoparticle inhibits mouse colitis by activating dendritic cell AMP-activated protein kinase. Mol. Ther..

[242] Wang F., Yuan M., Shao C., Ji N., Zhang H., Li C. (2023). Momordica charantia-derived extracellular vesicles provide antioxidant protection in ulcerative colitis. Molecules.

[243] Wang B., Guo X.-J., Cai H., Zhu Y.-H., Huang L.-Y., Wang W., Luo L., Qi S.-H. (2022). Momordica charantia-derived extracellular vesicles-like nanovesicles inhibited glioma proliferation, migration, and invasion by regulating the PI3K/AKT signaling pathway. J. Funct. Foods.

[244] Park Y.S., Kim H.W., Hwang J.H., Eom J.Y., Kim D.H., Park J., Tae H.J. (2023). Plum-derived exosome-like nanovesicles induce differentiation of osteoblasts and reduction of osteoclast activation. Nutrients.

